# Horizontal gene transfer from genetically modified plants - Regulatory considerations

**DOI:** 10.3389/fbioe.2022.971402

**Published:** 2022-08-31

**Authors:** Joshua G. Philips, Elena Martin-Avila, Andrea V. Robold

**Affiliations:** Office of the Gene Technology Regulator, Canberra, ACT, Australia

**Keywords:** horizontal gene transfer, lateral gene transfer, vertical gene transfer, GM plants, GMO risk analysis, gene technology regulation

## Abstract

Gene technology regulators receive applications seeking permission for the environmental release of genetically modified (GM) plants, many of which possess beneficial traits such as improved production, enhanced nutrition and resistance to drought, pests and diseases. The regulators must assess the risks to human and animal health and to the environment from releasing these GM plants. One such consideration, of many, is the likelihood and potential consequence of the introduced or modified DNA being transferred to other organisms, including people. While such gene transfer is most likely to occur to sexually compatible relatives (vertical gene transfer), horizontal gene transfer (HGT), which is the acquisition of genetic material that has not been inherited from a parent, is also a possibility considered during these assessments. Advances in HGT detection, aided by next generation sequencing, have demonstrated that HGT occurrence may have been previously underestimated. In this review, we provide updated evidence on the likelihood, factors and the barriers for the introduced or modified DNA in GM plants to be horizontally transferred into a variety of recipients. We present the legislation and frameworks the Australian Gene Technology Regulator adheres to with respect to the consideration of risks posed by HGT. Such a perspective may generally be applicable to regulators in other jurisdictions as well as to commercial and research organisations who develop GM plants.

## 1 Introduction

Horizontal or lateral gene transfer (HGT) is the stable and heritable acquisition by an organism, of genetic material that did not originate from a parental donor (Keese, 2008). Any DNA sequence, including endogenous sequences or foreign DNA introduced into a genetically modified (GM) organism, has the potential to undergo HGT. This potential is only fulfilled when the genetic material stably integrates into the genome of the recipient and is then transmitted to its offspring (Hülter and Wackernagel, 2008; Brigulla and Wackernagel, 2010; Huang, 2013). HGT can benefit the recipient by enabling the acquisition of a beneficial pre-existing trait from another organism, regardless of phylogenetic distance. It thereby, like vertical gene transfer, accelerates evolution (Fournier et al., 2015).

In Australia, the Gene Technology Regulator (the Regulator) receives applications for the intentional environmental release of GM plants and, as part of the assessment process of these applications, must consider the risks to human and animal health and to the environment from gene technology posed by the proposed activities. GM plants may have genetic elements sourced from other organisms imparting desired traits, e.g., increased nutritional value; drought, pest and disease resistance; or increased productivity. While gene transfer is most likely to occur to sexually compatible relatives through vertical gene transfer, the likelihood of gene transfer to non-sexually compatible organisms *via* HGT also needs to be considered as part of the risk assessment.

Similarly, in Europe, the Commission Regulation (EU) 503/2013 of 3 April 2013 (on applications for authorisation of genetically modified food and feed) states that “The applicant shall assess the probability of horizontal gene transfer from the product to humans, animals and microorganisms and any potential associated risk when intact and functional nucleic acid(s) remains in the genetically modified food and feed*.*” In the United Kingdom, the independent Advisory Committee of Releases into the Environment (ACRE) also considers HGT in their assessment for application for the release of genetically modified organisms (GMOs) (ACRE, 2013). Other regulatory authorities may also need to consider HGT before issuing an authorisation or licence. In this review, we discuss the recent advances in detecting HGT events and present updated evidence of the likelihood, factors, barriers and pathways for HGT to take place from GM plants to a variety of other organisms.

## 2 Legislative context and risk analysis applicable to considering risks imposed by HGT

**FIGURE 1:**
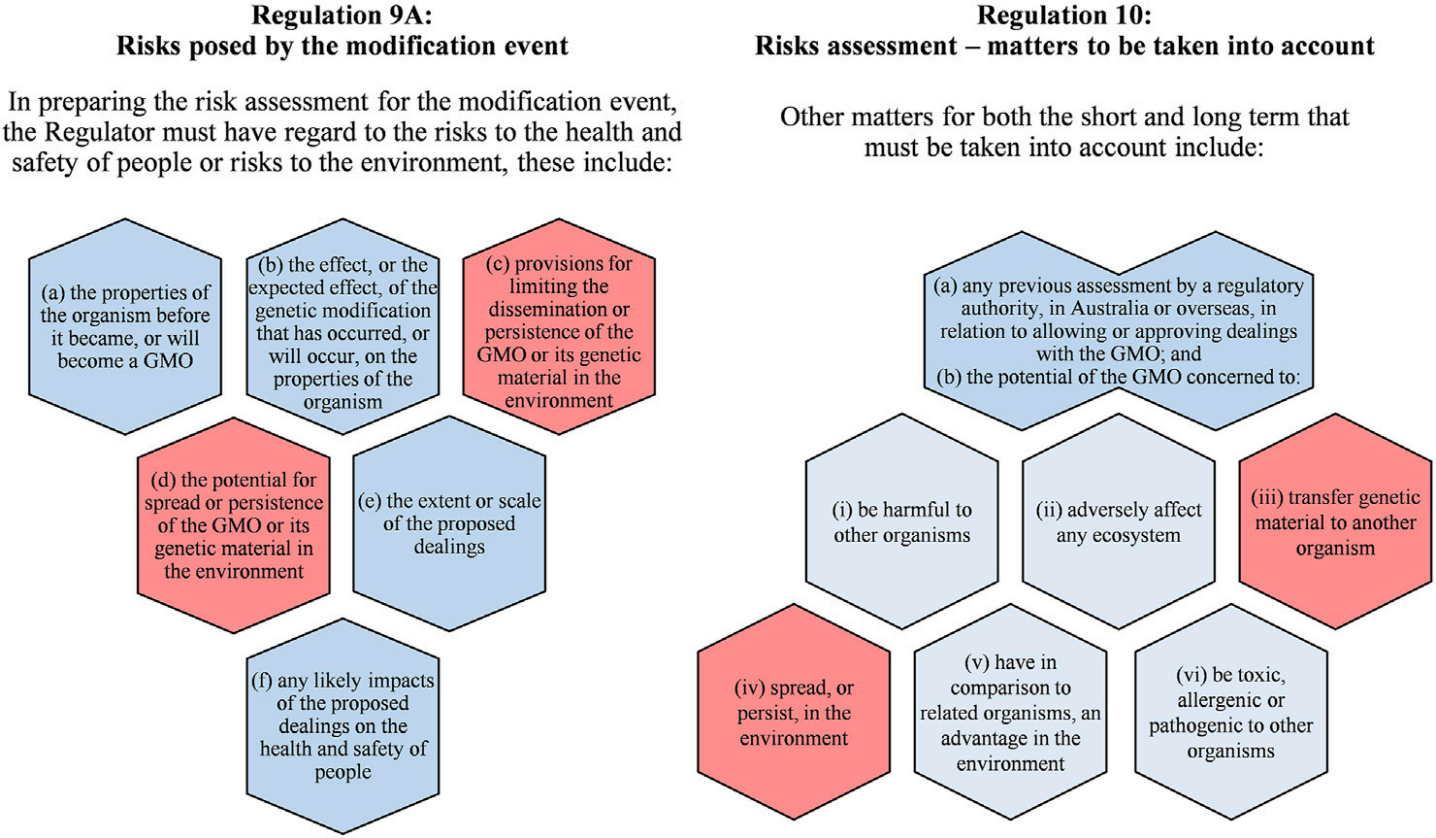
Summary of matters prescribed in the Australian Gene Technology Regulations 2001 that the Australian Gene Technology Regulator must consider in the risk assessment for a proposal to release a genetically modified (GM) organism, including a (GM) plant, into the environment (OGTR, 2020). Prescribed matters with a background shading of red may include consideration for horizontal gene transfer.

In Australia, Regulations 9A and 10 of the Gene Technology Regulations 2001 (OGTR, 2020) specify the risks and matters that must be considered in the risk assessment for an environmental release of GMOs (Figure 1). Considerations relating to gene flow are 1) the potential for spread and persistence of a GMO’s genetic material in the environment and 2) the potential of the GMO to transfer genetic material to another organism. The risk assessment seeks to evaluate the level of risk from the activities with a GMO if HGT from the GMO into other organisms was successful, compared to the status quo.

In Australia, the Risk Analysis Framework (RAF) (OGTR, 2013), in accordance with Australia’s *Gene Technology Act 2000* and Regulations, outlines the approach that the Regulator takes to conduct the risk assessment of proposed activities with a GMO. Such activities include the proposed environmental release of the GMO. The RAF describes the three essential components needed for a scenario (set of circumstances) that might give rise to harm as a result of activities conducted with the GMO. The three components are: 1) a source of potential harm, which may be a new or altered property/trait of the GMO; 2) a potential harm to people or the environment; and 3) a plausible causal linkage between components 1) and 2). If a plausible causal linkage or potential harm cannot be described, then the source of potential harm poses no risk (OGTR, 2013).

While HGT per se is not considered a risk, it fits into the pathway component of risk as it can link the introduced or modified DNA to a potential harm to people or the environment. Therefore, the likelihood of HGT occurring determines the potential for any harm. As the number of steps in a pathway leading to harm increases, the likelihood of harm occurring decreases. The Regulator considers the likelihood of occurrence of HGT and the severity of adverse outcomes if HGT was successful. If the level of risk is increased compared to the status quo, then the Regulator may include specific risk management measures to interrupt steps in a pathway and reduce the likelihood of harm occurring or refuse to approve the proposed intentional environmental release. For example, as a measure to limit the likelihood of gene flow by vertical gene transfer from GM plants, an exclusion zone can be imposed where sexually compatible plants are not permitted to be grown.

It should also be noted that risk analysis on a proposal for the release of GM plants occurs in the context of the receiving environment. For example, if a GM (transgenic) DNA sequence was sourced from a ubiquitous bacteria or fungi, then this forms part of the context as the DNA sequence is already in the environment. If HGT from the bacteria or fungi is more likely than HGT from the proposed activities with the GM plant, then the potential of HGT resulting in harm from the release of the GM plant can be no greater than the risk from the parent organism. Similarly, the likelihood of harm occurring as a result of the GM plant release is compared to that of harm occurring in the absence of the genetic modification, i.e., the non-GM plant.

## 3 Advances and limitations of new HGT detection methods

Until recently, comparative analysis for detection of HGT events relied on very limited databases of manually annotated genes (Dupont and Cox, 2017). However, the expansion of next generation sequencing has allowed an ever-increasing number of whole genomes from a vast range of species to be readily available for multiple genome comparisons. As a result, detection of HGT events for all annotated genes can now be performed bioinformatically. By using this approach, HGT can be inferred by either the parametric or phylogenetic methods (Ravenhall et al., 2015). The parametric methods look for sections that significantly differ from the average composition; these include GC content and codon usage (Ravenhall et al., 2015). The phylogenetic methods compare the evolutionary histories of a gene of interest and its homologues across multiple species, to identify conflicting phylogenies (Ravenhall et al., 2015; Soucy et al., 2015; Wybouw et al., 2018).

Advances in computational algorithms have also helped in the identification of additional HGT events from genomes that were previously analysed. For example, Wybouw et al. (2018) using refined bioinformatics parameters, identified 25 additional horizontally transferred genes in the spider mite (*Tetranychus urticae*) genome, seven years after HGT was first analysed in this species (Grbic et al., 2011). In the grass species, *Alloteropsis semialata*, initially two genes were identified to be horizontally transferred. Using next generation genome resequencing and strict phylogenetic comparisons amongst 146 other grass species, Dunning et al. (2019) were able to detect 57 additional horizontally transferred genes, some of which are associated with disease resistance and abiotic stress response loci (Dunning et al., 2019). In another example, ToxA, a fungal virulence protein, which is associated with diseases in wheat and barley was shown to reside on a horizontally transferred genomic cluster. The presence of this virulence protein provides a selective advantage to the fungus. Using long-read sequencing technologies, with the available genomes of fungal pathogens in the Pleosporales order, McDonald et al. (2019) were able to confirm the HGT origin of ToxA and define the boundaries of the transferred genomic cluster.

Other examples of HGT events that have been identified between species, using both bioinformatics and experimental methods, include: HGT of genes originating from bacteria, fungi, and plants to bdelloid rotifers (Gladyshev et al., 2008); HGT of carotenoid genes from fungi to pea aphids, causing a red colour polymorphism that provides selective advantage to avoid parasitism compared to green aphids (Losey et al., 1997; Moran and Jarvik, 2010); HGT of a photoreceptor gene from hornworts to ferns, allowing ferns to thrive under low-light conditions (Li et al., 2014); HGT from bacteria and fungi to silkworms of genes thought to confer disease resistance, nutrient and energy metabolism and toxin degradation (Zhu et al., 2011); HGT of an antifreeze protein gene between fish living in icy seawater (Graham et al., 2008), which the authors propose to naturally occur by sperm-mediated HGT during external fertilisation, where the sperm “absorbs” “naked” DNA from the environment; HGT of mitochondrial DNA from the parasite *Trypanosoma cruzi* to humans (Hecht et al., 2010); and HGT from humans to the strictly human pathogen *Neisseria gonorrhoeae* (Anderson and Seifert, 2011). Although far less frequent than HGT between bacteria, HGT from bacteria to eukaryotes has also been described, including the transfer of genes from the bacterium Wolbachia to insects and nematodes (Dunning Hotopp et al., 2007; Nikoh et al., 2008), from bacteria and fungi to plant parasitic nematodes (Noon and Baum, 2016) and from *Agrobacterium* to plants (Matveeva and Otten, 2019). It is to note that the aforementioned HGT examples have non-neutral or advantageous impacts, see section 5 below. A horizontally transferred gene is unlikely to be maintained in a population if it has a negative impact in the recipient.

Given the automated nature of genome data collection and gene prediction annotation in the contemporary setting, it is impractical to manually validate all genes within a genome. Therefore, concerns relating to whether the inferred HGT events in eukaryotes are statistically supported have been raised (Dupont and Cox, 2017). In addition, the short reads produced by many modern sequencing platforms raises concerns about microorganism contamination, especially involving the putative HGT between these microorganisms (Boothby and Goldstein, 2016; Wickell and Li, 2019). Even though new techniques allow HGT in eukaryotic genomes to be detected with greater frequency than a few years ago, HGT in complex eukaryotes is relatively rare when compared with the observed rates in simpler organisms such as viruses or prokaryotes (Andersson, 2005; Keese, 2008; Vogan and Higgs, 2011; Crisp et al., 2015; Qiu et al., 2016; Sieber et al., 2017).

## 4 Pathway considerations for HGT from GM plants

Direct or vector-mediated pathways can facilitate HGT. In direct pathways, the recipient organism either “takes up” DNA from another living cell or uptakes “free” or “naked” DNA present in the environment. Vector-mediated pathways are those where DNA is first taken up from the donor by an intermediate recipient that acts as a vector, such as a virus or prokaryote, and then passed on to a different recipient.

There are a number of factors that affect the likelihood of the introduced or modified DNA sequences in GM plants being successfully horizontally transferred and then retained in the final recipient. These include: the proportion of introduced DNA in the GM plant as a source for HGT; the availability and integrity of the introduced DNA sequence; the physical proximity of introduced DNA and a potential recipient organism; whether the recipient organism has a dedicated mechanism for uptake of DNA; whether homologous DNA sequences are present in the recipient organism; whether the donor and recipient are genetically compatible; and whether the horizontally transferred GM DNA sequence gives an advantage to the recipient organism. These factors will be discussed in the following sections.

### 4.1 Proportion of introduced DNA in GM plants

The likelihood of HGT of the introduced or transgenic DNA from GM plants to other organisms depends on its proportion in relation to the amount of total plant DNA. This proportion can be calculated when both the size of the transgenic insert and the size of the unmodified plant genome are known. As the proportion of the introduced transgenic DNA increases relative to the unmodified genome, so does the likelihood for it to be horizontally transferred.

Crops with single transgenic events, which have been approved for commercial release in Australia, such as altered fatty acid content safflower (GOR-73226-6 and GOR-7324Ø-2) and Roundup Ready™ canola (MON-ØØØ73-7) possess approximately 8.0 kb and 5.05 kb of transgenic DNA in a genome of approximately 2.75 and 1.13 Gb, respectively (Garnatje et al., 2006; Schreiber et al., 2018; Biosafety Clearing-House, 2019). Thus, the transgenic DNA component would account for approximately 0.00029–0.00045% of the total DNA in these crops.

Commercially released crops with stacked transgenic events, such as the six-stacked Agrisure^®^ Duracade™ 5222 corn^1^ (SYN-Ø53Ø7-1 × SYN-IR6Ø4-5 × SYN-BTØ11-1 × DAS-Ø15Ø7-1 × MON-ØØØ21-9 × SYN-IR162-4) and four-stacked Bollgard^®^ III × Roundup Ready™ Flex™ cotton (SYN-IR1Ø2-7 × MON-15985-7 × MON-88913-8 × MON 887Ø1-3) possess approximately 40 kb and 30 kb of transgenic DNA, respectively (Biosafety Clearing-House, 2019). With a genome size of corn and cotton at approximately 2.4 Gb (Schreiber et al., 2018), the transgenic DNA would account for approximately 0.0013–0.0017% of total DNA. Therefore, in these stacked event examples, the transgenic DNA occupies a greater proportion of the total crop DNA, and as such an increase in the likelihood of HGT, compared to GM crops with a single transgenic event. That stated, this proportion would be reduced in plants with a larger size genome, such as bread wheat with approximately 17 Gb (Schreiber et al., 2018), than in the previously mentioned stacked transgene examples.

While these GM crops provide examples where the transgenic DNA is introduced at a low copy number into the nuclear genome, other options include introducing transgenic DNA into the mitochondrial or chloroplast genome of plants. Depending on the plant tissue, multiple mitochondria and chloroplast organelles are present within an individual plant cell. Typically, 10 s–100 s of these organelles are present in *Arabidopsis* and tobacco leaf cells (Maliga and Bock, 2011; Sakamoto and Takami, 2018; Shen et al., 2019), with each organelle possessing multiple copies of its genome (Sakamoto and Takami, 2018). For example, if transgenic DNA was introduced into chloroplasts, 100 s–1000 s of copies of the gene are likely to be present per leaf cell (Pontiroli et al., 2010; Sakamoto and Takami, 2018), thereby increasing the proportion of transgenic DNA in the GM plant. Overall, the proportion of transgenic DNA in GM plants, both with single and stacked transgenic events, currently authorised for environmental release in Australia represents a minute fraction of the total plant genome.

### 4.2 Availability and integrity of DNA for HGT

#### 4.2.1 DNA in living plant cells

Plants are frequently exposed to harmful UV radiation, physical shearing and other forces that can damage and alter their DNA. However, DNA in living plant cells is protected through a variety of checking and repair mechanisms. These processes ensure that DNA is maintained to a very high integrity (reviewed in Bray and West, 2005). If the integrity of DNA is not maintained, DNA fragmentation could occur. Should fragmented DNA be horizontally transferred to another organism, it is unlikely to encode a functional protein product. All plant DNA, whether originating in the nucleus, mitochondrion, or chloroplast, is compartmentalised to their respective organelles. In addition to the plant cell wall, this compartmentalisation serves as a physical barrier to limit the availability of DNA to be horizontally transferred from living cells. These physical characteristics would be the same for both GM and non-GM plant DNA.

#### 4.2.2 Naked DNA

When the DNA is no longer contained within cells it is known as “free” or “naked” DNA. Naked DNA is accessible to microorganisms which possess mechanisms to uptake it from their surroundings. Such DNA can arise when: 1) plants deliberately release extracellular DNA, e.g. from their root tips as a defence strategy against soil microbial pathogens (Hawes et al., 2012); and/or 2) after cell death or damage, where the DNA is no longer protected by cell components and is released due to cellular degradation.

The integrity of naked DNA depends on many biotic and abiotic factors (Pontiroli et al., 2007) and most naked DNA is degraded within hours to weeks due to the adverse influence of the surrounding environment. However, small amounts of naked DNA may associate with smaller substrate particle sizes, such as minerals in sand and clay, thereby increasing DNA survival and therefore its availability for HGT (see review by Sand and Jelavić, 2018). It is worth noting that DNA fragments of any size can be internalised by competent prokaryotes and may become incorporated into their genome.

Examples of testing for persistence of naked transgenic DNA are available in the literature. In one experiment the fate of GM transplastomic tobacco DNA and the likelihood of HGT under ideal environmental conditions was investigated. Here the antibiotic resistance gene, *aadA*, which is commonly found in soil bacteria was inserted into the DNA of chloroplasts (Pontiroli et al., 2010). Non-GM and GM tobacco leaf tissue (0.05 g or 0.5 g; whole and ground) and purified GM tobacco DNA were placed into test tubes containing soil and maintained for approximately 4 years. After 4 weeks the amount of total DNA recovered was similar across all samples, however, only 0.002% of total plant DNA was recovered after 4 years. With respect to the transgene, the number of *aadA* gene fragments decreased by more than 10^4^-fold over the first 2 weeks, and then by a further 10-fold over the remainder of the experiment. Furthermore, extracted DNA from the soil treatments was transformed into *Acinetobacter* modified to facilitate homologous recombination. Transformed *Acinetobacter* were obtained using total DNA from soil samples containing purified GM tobacco DNA at 0 weeks, but not at later time points. In GM leaf samples, transformants were only obtained using DNA from soil samples that were supplemented with ground 0.5 g of GM leaf discs, but not other leaf treatments.

In another experiment, 2 years after GM sugar beets were harvested, shredded, and disposed, transgenic DNA was detected by PCR in the soil from the disposal site (Gebhard and Smalla, 1999). Although these examples may demonstrate that naked DNA (either intact genes or fragments) can survive for long periods of time, it is currently unknown what this length of time is and the percentage of DNA that would become fragmented. That said, transgenic DNA has the same physical properties as endogenous DNA, resulting in the same likelihood of transgenic DNA being horizontally transferred as that of non-GM plant DNA. A small percentage of naked DNA may therefore be available for HGT not only across time, but also across space as soils and sediments are subject to geological events (noting the degrading effects of abiotic and biotic interactions on DNA integrity).

### 4.3 Dedicated DNA uptake mechanisms in potential recipients for GM plant DNA

HGT can either occur through a vector-mediated pathway, such as *via* bacteria, viruses, viroids, plasmids or transposons, or *via* a direct pathway, such as exchange and uptake of naked DNA. HGT is most prominent in prokaryotes, especially in bacteria, who utilise it as a mechanism for adaptation, particularly for the acquisition of beneficial traits such as antibiotic resistance when placed under selective pressures (Soucy et al., 2015). HGT in prokaryotes usually occurs *via* conjugation, transformation and transduction. Other mechanisms for HGT include: prokaryotic cell fusion, exchange *via* gene transfer agents, intracellular or endosymbiotic gene transfer, which predominantly pertains to eukaryotes, and introgression. These vast array of mechanisms are thoroughly reviewed in a number of publications, e.g., Soucy et al. (2015) and De Santis et al. (2018).

#### 4.3.1 Conjugation

HGT *via* conjugation requires the physical association between the donor and the recipient cell. A well-characterised conjugation system occurs between *Agrobacterium* and plants. *Agrobacterium* sp. are soil-based plant pathogens that possess a type IV secretion system (T4SS), allowing the natural transfer and integration of part of its DNA, known as transfer-DNA, or T-DNA to the plant genome (Gelvin, 2017). The presence of historical HGT taking place from naturally occurring *Agrobacterium* has been described in sweet potato (Kyndt et al., 2015), in several *Nicotiana* species (reviewed by Chen and Otten, 2017) and recently in banana and over 30 dicot species, including commonly consumed foods such as peanuts, walnuts, guava, hops (used in beer production) and tea (*Camellia sinensis*, which is used for most teas) (Matveeva and Otten, 2019). In a process known as *Agrobacterium*-mediated transformation, biotechnologists ‘disarm’ the natural genes on the T-DNA and transform the *Agrobacterium* with a plasmid containing transgenes of interest. As the T4SS can act in *trans*, this modified *Agrobacterium* can be used as a vector to produce GM plants (Gelvin, 2003). However, both biotechnologists and gene technology regulators need to consider genetic elements outside the T-DNA, such as those on the *Agrobacterium* chromosome (Ülker et al., 2008) or on mobile genetic elements (Philips et al., 2017), which in some cases, have also been shown to be horizontally transferred into the plant genome during the *Agrobacterium*-mediated transformation process.

#### 4.3.2 Transformation

Transformation provides another mechanism for HGT, whereby naked DNA is “taken up” from the environment by naturally competent cells, which are predominantly bacteria (Blokesch, 2016). It has been shown under laboratory conditions that approximately 1% of bacterial species can take up DNA from the environment (Mao and Lu, 2016). Transformation can occur in environments where the donor or the intact donor DNA and the receiving organism are in close proximity. With respect to GM plant material, such environments include, but are not limited to, the gastrointestinal tract (GIT) of consumers and the plant phytosphere, which is a complex plant micro-ecosystem comprising of both the exterior and interior of plants that are aboveground and belowground (Yang et al., 2013).

#### 4.3.3 Transduction

Transduction is a process whereby bacteria and archaea acquire DNA *via* HGT, and this process is mediated through phages (Soucy et al., 2015). Transduction can be either generalised or specialised. In generalised transduction, a random piece of the host DNA is incorporated by the phage during lytic phage replication in place of the viral genome. In specialised transduction, an integrated prophage imprecisely excises itself from a host genome and incorporates some of the flanking host DNA (Soucy et al., 2015; Schneider, 2017). These “mistakenly” packaged host DNA can then be horizontally transferred *via* phages to the next bacterium and are likely to occur in environments where phages and bacterium are abundant, such as in waterways and the human GIT (Schneider, 2017).

#### 4.3.4 Gene transfer agents

Gene transfer agents (GTAs) are phage-like particles, found in bacteria and archaea, that can randomly incorporate a piece of the donor’s host genome for delivery upon cell lysis to other nearby recipient hosts and as such, can also facilitate HGT (Lang et al., 2012; Soucy et al., 2015). However, GTAs have lost their ability to target their own DNA for packaging. Therefore, they cannot transfer all the genes needed to encode their particle in the new recipient host, creating a distinction from phages participating in transduction (Lang et al., 2012; Soucy et al., 2015).

### 4.4 Homologous DNA sequences and genetic compatibility

The phylogenetic relationship between the donor and the recipient could also be a major determinant for HGT frequency. Despite the fact that all organisms have a history of HGT (Keese, 2008; Crisp et al., 2015; Fournier et al., 2015), the phylogenetic distance between non-related organisms increases the possibilities of genetic incompatibility, making them less likely to undergo HGT when compared with closely related organisms with compatible genomes (Bertolla and Simonet, 1999; Keese, 2008; Boto, 2010; Hibdige et al., 2021). Conversely, there is a greater likelihood of HGT if homologous regions are present between the donor and recipient (de Vries et al., 2001). Such homologous regions are more likely to be present in closely related organisms, such as between bacteria (Soucy et al., 2015). HGT between bacteria occurs frequently (McAdams et al., 2004). However, based on experimental data, HGT from purified DNA or ground GM plant tissue material to bacteria that lack flanking homologous DNA regions has also been shown to occur. Estimates indicate that this event occurs at a low frequency of 7 × 10^–23^ per cell. This frequency, as expected, increases if short homologous DNA sequences are present between the donor (GM plant material) and recipient bacteria (7 × 10^–13^ per cell), but it is still a few orders of magnitude (10^14^) lower to the naturally occurring rates of HGT between bacteria in the environment (10^–1^ to 10^–8^ per cell) (Dröge et al., 1998; Brigulla and Wackernagel, 2010).

Transgenes which have not originated from plants are generally codon optimised for improved expression when introduced into the GM plant. Prominent examples of bacterial transgenes that have been codon optimised and used in GM plants include the *cry* genes from *Bacillus thuringiensis* imparting insect resistance (Latham et al., 2017) and the *CP4 epsps* gene from *Agrobacterium* sp. strain CP4 imparting resistance to the herbicide glyphosate (Heck et al., 2003). Codon optimisation can reduce the likelihood of GM plant to bacteria HGT due to the reduction in homology between the optimised transgene and endogenous bacterial sequences. Additionally, if HGT of the intact codon optimised transgene to bacteria were to take place, the encoded protein product would be sub-optimal in expression and may not be retained within the population.

### 4.5 Proximity of donor DNA to a potential recipient organism

The proximity between the recipient and the donor or the donor’s intact DNA is another factor in the likelihood of HGT being successful. Therefore, the relationship between a donor and a symbiont, commensal, epiphyte, pathogen, predator or pest, that facilitate a close physical contact, increase this likelihood (Rumpho et al., 2008; Nikoh and Nakabachi, 2009; de la Casa-Esperón, 2012; Soanes and Richards, 2014; Qiu et al., 2016; Yin et al., 2016; Shinozuka et al., 2017). For plants, the micro-ecosystem comprised by the phytosphere is considered a hotspot for HGT between plants and bacteria (Pontiroli et al., 2009).

Wastewater treatment facilities, where wastewater from a variety of sources, including municipalities, hospitals, and industry converge, are also potential hotspots. This potential is due to the close contact of microorganisms from the variety of different sources, which may form biofilms and the selective pressures caused by pollutants such as heavy metals and antibiotics that can promote HGT (Hultman et al., 2018). The potential for HGT from GM plants to bacteria in wastewater treatment facilities in United States has been described (Gardner et al., 2019).

With respect to GM plants, other hotspots include the GITs of animals and humans after GM plant consumption. For example, the human GIT provides an excellent environment for HGT, with its stable physicochemical conditions and temperature, continuous food supply, high concentration of bacteria and their bacteriophages, and plenty of opportunities for conjugation on the surfaces of food particles and host tissues (Lerner et al., 2017). During the digestive process, consumed food is broken down and fragments of DNA are released in the GIT (see section 4.6.3 for HGT to bacteria in the gastrointestinal tract of humans and animals) and become available for transformation by naturally competent cells. In addition to co-localisation of GM plant DNA and the potential recipient, sufficient time needs to be available for HGT to take place. Bacteria are considered the most likely recipients of HGT from GM plants, because they possess several mechanisms facilitating DNA uptake (see section 4.3 above) and they have many opportunities to form close physical proximity with plants and/or their DNA.

The following sections will describe the likelihood, factors and the barriers for HGT to take place from plants, including GM plants, to a variety of recipients.

### 4.6 HGT from plants to bacteria

#### 4.6.1 HGT to bacteria in the phytosphere

Despite the fact that potential recipients for transgenic DNA have been identified among soil bacteria (Monier et al., 2007), there is no evidence in the published literature of HGT from a GM plant to soil bacteria under field conditions (Badosa et al., 2004; Demanèche et al., 2008; Ma et al., 2011). For example, the root-associated microbiota was studied in a field 6 years after planting with virus-resistant GM grapevine. In addition to a viral coat protein, the GM grapevine also possessed the *nptII* antibiotic resistance gene (conferring resistance to kanamycin) as a marker. The analysis showed that the presence of GM grapevine did not increase the level of *nptII*-resistant bacteria in the soil, as similar levels of naturally *nptII*-resistant bacteria were found in soil planted with non-GM grapevine (Hily et al., 2018).

#### 4.6.2 HGT to bacteria in aquatic environments

Similar to the considerations for naked DNA in the terrestrial environment, naked DNA in the aquatic setting also needs to remain intact for the likelihood of aquatic microorganisms to incorporate this DNA into their genome and then produce its functional protein product. The persistence of naked DNA in water samples (groundwater and river water) originating from GM corn (event MON-ØØ863-5) and purified plasmid DNA, both containing the *nptII* antibiotic resistance gene, was measured by the ability of *Pseudomonas stutzeri* to naturally take up the naked DNA (Zhu, 2006). The results, based on *P. stutzeri’s* natural uptake, showed the presence of the plasmid DNA in intact or filter sterilised water but that this decreased to undetectable levels within 4 days (Zhu, 2006), indicating that elements in these water samples aided DNA degradation. Likewise, in the same study, the stability of GM plant DNA was assessed by real-time PCR. The results demonstrated that the concentration of GM plant DNA reduced by two orders of magnitude within 4 days in intact and filter sterilised water (Zhu, 2006). Thus, material such as pollen, leaves, fruit and other plant detritus, originating from GM plants could potentially make its way to the aquatic environment and become available for HGT should its DNA maintain integrity (Poté et al., 2009).

#### 4.6.3 HGT to bacteria in the gastrointestinal tract of humans and animals

In their diets, humans and animals are regularly exposed to DNA from a variety of sources, including from plants, animals and microorganisms. Nawaz et al. (2019) reviewed that as part of a normal human diet, the daily dietary intake of DNA ranged between 0.1 and 1 g. The likelihood of HGT of transgenic DNA from GM plants to gut bacteria or tissues of animals and humans is very low when considering the total pool of all available DNA in the GIT (Jennings et al., 2003; Netherwood et al., 2004; Sieradzki et al., 2013; Korwin-Kossakowska et al., 2016). Estimates for the percentage of GM DNA in a theoretical Austrian daily diet were performed by Jonas et al. (2001). As part of their daily average diet, Austrians would consume 170 g of soybean, maize, and potato. Based on a total daily dietary DNA intake of 0.6 g, and considering the consumption of purely GM crops, approximately 0.00006% of the total DNA would be GM (Jonas et al., 2001). Similarly, in dairy cows consuming 60% GM maize, approximately 0.000094% of the total daily DNA intake would be GM (Beever and Phipps, 2001). However, these estimated percentages are based on intact DNA prior to consumption and have not considered the fate of the DNA during the digestive process. As the DNA from GM organisms, including GM animals, insects and plants is chemically equivalent to DNA from other sources, the fate of GM DNA in the GIT is similar to that of non-GM DNA (Rossi et al., 2005; Van Eenennaam and Young, 2014). This fate is purely for the DNA and there would be separate considerations for regulators for any consumed proteins encoded by the GM DNA, which is beyond the scope of this review.

There are several factors that detrimentally affect the integrity and availability of DNA. These include the process of food preparation, cooking, and digestion of DNA in the GIT, all of which fragment the DNA (these factors have been reviewed in Rizzi et al. (2012) and Nawaz et al. (2019)). Thus, only in rare circumstances is it likely that an intact gene or a transgene is able to participate in HGT from dietary sources to consumers or to the bacteria in their GIT.

In insects, the discovery of incompletely digested leaf fragments in the faeces of tobacco hornworm fed on GM transplastomic tobacco carrying the *nptII* gene raised the possibility that gut bacteria could uptake GM plant DNA (Deni et al., 2005). However, this could not be confirmed in the gut bacteria of the species tested so far, which include tobacco hornworm and bees (Deni et al., 2005; Mohr and Tebbe, 2007; Hendriksma et al., 2013; Niu et al., 2017). Experiments to investigate HGT to the bacteria in the GIT of birds and mammals have also been undertaken. For example, GM corn, GM rice, GM soybean or purified plasmid DNA were introduced in the diets of rats, broilers, laying hens, pigs, piglets and calves. No instances of HGT of the introduced DNA to bacteria in the GIT was observed in these experiments (Nemeth et al., 2004; Zhu et al., 2004; Deaville and Maddison, 2005; Calsamiglia et al., 2007; Wilcks and Jacobsen, 2010; Yonemochi et al., 2010; Walsh et al., 2011; Nordgård et al., 2012; Sieradzki et al., 2013; Świątkiewicz et al., 2013; Zhao et al., 2016).

Overall, HGT from GM plants to bacteria has rarely been reported (Nielsen et al., 1998; Andersson, 2005; Pontiroli et al., 2009; Miyashita et al., 2013). This is likely to be a consequence of the small percentage of introduced DNA in GM plants (see section 4.1 above) combined with the low HGT frequency from plants to prokaryotic recipients (Pontiroli et al., 2009; Brigulla and Wackernagel, 2010).

### 4.7 HGT from plants to eukaryotes

#### 4.7.1 Direct HGT to humans and animals

Animals are multicellular eukaryotes whose cells lack walls. Most animals cannot synthesise their own nutrients, but instead rely on obtaining these by digesting other organisms as food. If plant material is consumed, its DNA will be present in the animal’s GIT (Broaders et al., 2013). For HGT to become a reality, the consumed DNA would have to maintain its integrity after digestion, be horizontally transferred to the reproductive cells and then be passed on to the recipient’s offspring. This could be achieved by consumed DNA being transferred into germline cells either directly, if these are physically close to the digestive system, or *via* a circulation system, such as the blood in vertebrates, or the haemolymph in lower animals.

The presence of GM DNA in a variety of higher animals who have consumed GM plants as part of their diet have been tested. For example, herbicide tolerant Roundup Ready^®^ soybean (event MON-Ø4Ø32-6) and insect resistant corn (event MON-ØØ81Ø-6) were used in feeding experiments that were carried out over ten generations on Japanese quails. The results showed no signs of GM DNA in tissue samples, including the breast muscle, eggs and internal organs (Korwin-Kossakowska et al., 2016). In other studies, fragments of ingested DNA have been detected in the blood of humans and a variety of higher animals, which have been extensively reviewed (e.g., Parrott et al., 2010; Nicolia et al., 2014; Nadal et al., 2018). Small amounts of fragmented DNA have also been shown to be absorbed into the gut epithelial tissues of mammals (Rizzi et al., 2012). It is to note that fragmented DNA may no longer be able to encode a protein in its entirety and as such is unlikely to be functionally active. In other reports, small fragments of GM DNA have been detected in some tissue samples from pigs, sheep and birds (Jennings et al., 2003; Mazza et al., 2005; Rossi et al., 2005; Sharma et al., 2006). Albeit present in some tissues, there was no evidence of GM DNA integration into the genome of somatic cells, or its transfer into the germ cell DNA in these animals.

In addition, there has been no GM DNA or protein detected in consumed products such as milk, meat or eggs from livestock that have been with fed GM plants (reviewed by Van Eenennaam and Young, 2014; De Santis et al., 2018). Nawaz et al. (2019) suggest that uptake of fragmented DNA into the bloodstream of consumers is a common occurrence. Testing in these studies was generally conducted within 24 h after consumption and detection of the ingested DNA was most likely to originate from high-copy-number genes, such as those present in the chloroplast (reviewed in Nadal et al., 2018; Nawaz et al., 2019). However, after 24 h, the ingested DNA present in the blood was difficult to detect, indicating that there are mechanisms in place to eliminate them (Nawaz et al., 2019).

An important consideration for multicellular eukaryotes is that the horizontally acquired genes would need to reach the germ line and then be transferred to the next generation. This entails an extra barrier for HGT and chances of transmission of horizontally transferred genes to offspring are rare, even if transmission happens during unicellular or early developmental stages (Huang, 2013). In an alternative pathway, a fetus may be exposed to DNA fragments when the pregnant mother consumes DNA-containing material. In studies conducted in the late 1990s, pregnant mice were fed with high levels of purified phage M13 and plasmid DNA. The presence of this foreign DNA was then tested in the fetuses and in new-born mice, where fragmented phage M13 DNA was detected (Schubbert et al., 1997; Schubbert et al., 1998). The results showed that not all cells in the fetuses or new-born mice contained this foreign DNA. It was concluded that the DNA fragments were most likely transferred across the placenta from the mother. It was not clear if DNA fragments integrated into the genome of the somatic cells of the offspring or if they were present transiently. The limitations and conclusions of this study have been extensively critiqued by Beever and Kemp (2000). A follow-up study by the original researchers found no transfer to the germline cells when mice were fed transgenic DNA daily over eight generations (Hohlweg and Doerfler, 2001).

**FIGURE 2:**
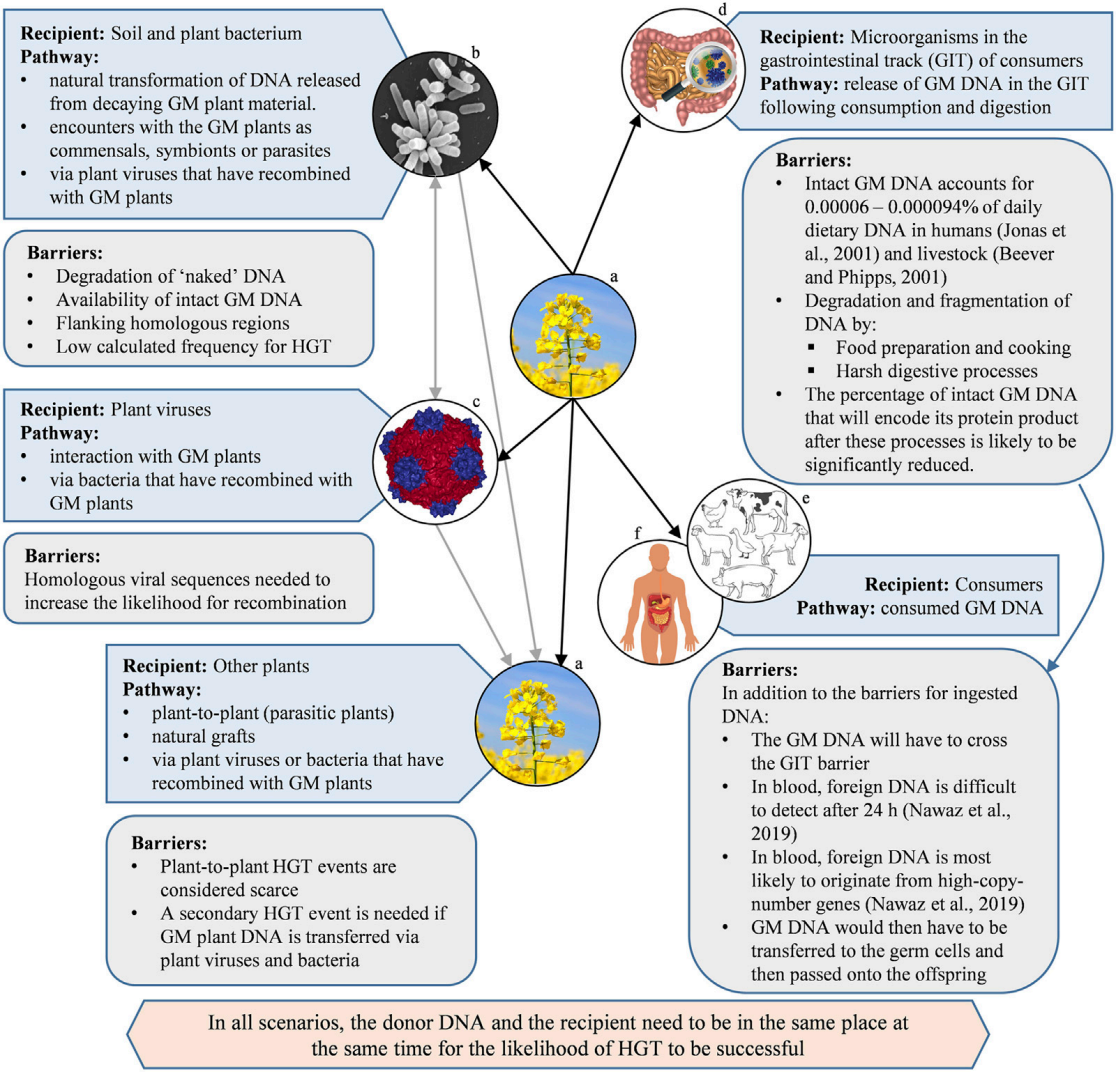
A selection of pathways and barriers for the horizontal gene transfer (HGT) from (GM) plants to a selection of recipients. Black arrows indicate direct HGT, grey arrows indicate a secondary HGT event from plant viruses and/or bacterium. [Images courtesy of: **(A)** canola by Pixabay/Jenő Szabó, **(B)** *Agrobacterium* by Jing Xu, Indiana University, **(C)** Cowpea mosaic virus, PDB ID: 1NY7 (Lin et al., 1999), created using NGL viewer (Rose et al., 2018) at RCSB PDB, **(D)**, **(E)** and **(F)** sourced from iStock].

In summary, if an animal diet includes the consumption of GM plants, there are several barriers that need to be overcome for HGT of the transgene to take place (Figure 2). These include: The extremely low percentage of the GM DNA in the overall dietary intake, which is chemically equivalent to non-GM DNA; the fragmentation of DNA due to digestion, reducing the likelihood of the GM DNA to code for a functional product; the additional hurdle of the GM DNA crossing the gastrointestinal barrier; and persisting in the bloodstream for it to be available for incorporation into germline cells. If all these challenges could be overcome, and for HGT to be successful, the GM DNA would then have to be passed onto the consumer’s offspring.

#### 4.7.2 HGT between plants

Plants are multicellular eukaryotes, most of which are capable of synthesising their own nutrients. Plant cells possess a cell wall that is made up of cellulose, which adds a physical barrier in accessing the DNA within living cells or taking up DNA from other plants. The intimate contact required for HGT between plants may occur in natural or artificial grafts, or *via* parasitic interactions. During these interactions, an exchange of substances, including nucleic acids can occur. Although epiphytic plants are in contact with their host throughout their lives, there is only superficial contact with the surface of the host rather than tight interaction between the two. This is a barrier to direct HGT between epiphytes and their hosts.

##### 4.7.2.1 Direct HGT of nuclear plant DNA to other plants

The strongest evidence of plant-to-plant HGT occurring are those between parasitic plants and their hosts (Xi et al., 2013; Davis and Xi, 2015; Yang et al., 2016; Yoshida et al., 2016; Yang et al., 2019). This is most likely due to formation of a multicellular organ called the haustorium when the parasitic plant encounters the host. The haustorium creates an intimate physical association by penetrating into the host stem or root and then connecting to the host vasculature, which allows the exchange of a wide range of materials including DNA and RNA (Yoshida et al., 2016).

Some recent studies of HGT involving plants, predominantly in grasses, have been described. Such examples include: multiple HGTs of nuclear ribosomal genes between grass lineages (Mahelka et al., 2017); HGT between distantly related grasses of a second enzymatic gene that aids in microhabitat variation (Prentice et al., 2015); and evidence of the contribution of nuclear HGT to C_4_ evolution in grasses (Christin et al., 2012). More recently, genomes of a diverse set of 17 grass species that span more than 50 million years of divergence were analysed for grass-to-grass protein-coding HGT events. The results indicated that major crops, such as maize and wheat were recipients to horizontally transferred genes (Hibdige et al., 2021).

##### 4.7.2.2 Direct HGT of non-nuclear plant DNA to other plants

In general, HGT of non-nuclear DNA, i.e., mitochondrial and chloroplast DNA, between individual plants is considered to be more likely than nuclear DNA transfer due a variety of factors including; their high copy number and a process known as organelle capture (Stegemann et al., 2012). For example, in natural grafts, where two plant stems or roots are in contact with each other, or under laboratory grafting experiments, the transfer of entire chloroplast genomes or even full mitochondria organelles have been detected (Stegemann and Bock, 2009; Stegemann et al., 2012; Thyssen et al., 2012; Gurdon et al., 2016). However, some authors suggest that heritable changes might only be possible if the formation of lateral shoots occurs within the graft site (Stegemann and Bock, 2009), certainly heritable changes can be induced following grafting under laboratory conditions (Fuentes et al., 2014). As such, the stability of horizontally transferred genes *via* natural grafting (regarding integration, expression, and inheritability) requires additional analysis (Gao et al., 2014).

Transfer of non-nuclear DNA has also been shown to occur independently of grafting. For example, the whole genome analysis of *Amborella trichopoda*, which is thought to be the most basal extant flowering plant revealed six genome equivalents of historical horizontally acquired mitochondrial DNA. These were acquired from green algae, mosses, and other angiosperms and some transferred as intact mitochondria (Rice et al., 2013). Non-nuclear DNA transfer also occurs in parasitic interactions (Xi et al., 2013; Davis and Xi, 2015; Yoshida et al., 2016; Sanchez-Puerta et al., 2019; Sinn and Barrett, 2019) and mitochondrial HGTs in both directions have been detected in 10 of 12 parasitic lineages (Yoshida et al., 2016).

The number of chloroplasts per plant cell is highly variable, with approximately 100–120 chloroplasts per cell in the leaves of tobacco and *Arabidopsis* (Maliga and Bock, 2011). Thus, GM plants possessing the transgene in the chloroplast, for example, have a much higher transgene copy-number than nuclear-modified plants. In addition, as the chloroplast is prokaryotic in origin, it is more likely to share homologous regions with other prokaryotes. Therefore, transgenes within the chloroplasts of GM plants have been proposed to increase the likelihood of HGT to bacteria compared to transgenes integrated into nuclear DNA (Kay et al., 2002; Monier et al., 2007). However, studies comparing plasmid DNA, PCR products and chloroplast-transformed tobacco, containing ∼7,000 copies of the transgene per plant cell, all of which contained the same DNA construct concluded that there was no indication that these high-copy-number chloroplast transformed GM plants could cause higher rates of HGT than nuclear-transformed GM plants (Demanèche et al., 2011).

Currently, to the best of our knowledge, there have been no reports of HGT from a GM to a non-GM plant.

### 4.8 HGT from plants to viruses

Plant viruses could also be recipients of genes horizontally transferred from GM plants. Viruses frequently evolve by recombination between homologous viral sequences (Keese, 2008). Therefore, GM plants carrying virus-derived sequences, such as viral promoters, might be more likely to act as an HGT donor for plant viruses capable of infecting these GM plants (Keese, 2008).

An *in vivo* study of HGT from GM grapevine was carried out by assessing its root-associated microbiota 6 years after planting (Hily et al., 2018). The grapevine was modified by introducing the coat protein from *Grapevine fanleaf virus* (GFLV) strain F13 (*F13-cp*) to confer resistance against the virus as well as the *nptII* marker gene as previously discussed. For the viral transgene, analysis of the GFLV population showed a large number of natural recombination events within the virus; however, none of these recombinants contained the *F13-cp* or *nptII* transgene sequence (Hily et al., 2018).

Under laboratory conditions, plant viruses demonstrate the ability to incorporate plant DNA or RNA into their genome. For example, experiments were conducted with *Cucumber necrosis virus* (CNV), which is a positive-sense, single-stranded RNA virus. When *Nicotiana benthamiana* leaves were infiltrated with the transcript of CNV coat protein, virus-like particles were produced that carried a variety of host RNAs, including retrotransposons and chloroplast-specific RNAs (Ghoshal et al., 2015). In the case of retrotransposons, the authors concluded that it would be possible for these to be horizontally transferred *via* the virus to new hosts (Ghoshal et al., 2015). Likewise, the *Beet curly top Iran virus* (BCTIV), a single-stranded DNA virus, can incorporate DNA from its sugar beet host to form hybrid virus-plant minicircles. These can then be packaged and have been shown to replicate and be transcribed in other plant species sensitive to BCTIV infection (Catoni et al., 2018).

### 4.9 HGT from plants to other organisms and facilitation *via* vectors

The introduced DNA in GM plants has the potential to be horizontally transferred to other classes of organisms that have not been mentioned in this review, either through a direct or a vector-mediated pathway. Such organisms include, but are not limited to, algae, fungi, or nematodes. For example, rare HGT events from plants to fungi have been reported (Richards et al., 2009; Nikolaidis et al., 2013; Li et al., 2018). Should HGT from plants to recipients, such as fungi and bacteria take place, the possibility of the recipient itself acting as a secondary HGT donor/vector becomes available.

Other potential recipients include arthropods and nematodes, which also have a history of horizontally acquiring genes from bacteria and fungi (Mitreva et al., 2009; Haegeman et al., 2011; Wybouw et al., 2016). Similarly, viruses could act as a HGT vector facilitating gene transfer from plants to bacteria. However, viruses that function in both plants and bacteria are rare (Nielsen et al., 1998), although certain plant viruses, such as geminiviruses, have been shown under experimental conditions to replicate in the bacterium, *Agrobacterium tumefaciens* (Rigden et al., 1996). If HGT was to then take place between the virus and bacterium under field conditions, a secondary vector-mediated pathway could become available, with bacteria then acting as a HGT donor to other plants. Thus, in this scenario, HGT *via* two vectors could take place between GM and non-GM plants.

Likewise, bacteria could horizontally transfer DNA that it has acquired from GM plants *via* HGT to other organisms. However, these successive processes would most likely require several more barriers to be overcome, each reducing the likelihood for successful HGT, and would most likely need to be carried out over an evolutionary timescale.

## 5 Considerations regarding the potential for adverse outcomes as a consequence of HGT

As previously discussed, while the acquisition of a new gene by HGT is not considered harm per se, it has the potential to lead to genetic variation within a population, and thus, its impact on driving the evolutionary function of organisms has been considered (Keese, 2008; Boto, 2010). With respect to GM plants, for HGT-induced harm, the acquisition of the genetic material must result in a non-neutral change for the recipient, be maintained in the population and result in an adverse outcome to humans, animals and/or the environment (Keese, 2008).

Occasionally, the function of the transferred genes could strongly affect the severity of the adverse consequences or the likelihood of a HGT event (Keese, 2008). When a population is under strong selective pressures or environmental stresses, HGT can be stimulated. In the recipient, the transferred gene can either confer a detrimental, neutral, or advantageous trait. If this novel trait is advantageous, the recipient can overcome its pressures and stresses, outcompete its neighbours or adapt to a new ecological niche (van Elsas et al., 2003; Keeling, 2009; Raz and Tannenbaum, 2010; Vogan and Higgs, 2011; de la Casa-Esperón, 2012). For example, under intensive agricultural production, the coffee berry borer beetle, *Hypothenemus hampei*, horizontally acquired a mannanase gene from bacteria that helped it adapt to enzymatically digest the polysaccharides of coffee beans, converting it into an invasive pest (Acuña et al., 2012). Similarly, the whitefly, *Bemisia tabaci*, horizontally acquired a phenolic glucoside malonyltransferase gene from plants allowing it to neutralise the plant-produced phenolic glycosides that would otherwise kill the whitefly after herbivory. This to our knowledge is the first known example of a HGT event between a plant and an animal and is thought to have occurred ∼86 million years ago (Xia et al., 2021). There are also instances that illustrate how novel genes acquired by HGT contributed to parasite adaptation to a new host in different organisms, for example: HGT of cellulase genes, allowing cellulase activity, from several microbial donors to nematodes, which enhances their parasitism and pathogenicity of plants (Danchin et al., 2010); HGT of genes associated with disease resistance and abiotic stress response in spider mites (Grbic et al., 2011; Wybouw et al., 2018); and HGT of fungal genes involved in the metabolism of plant sugars and genes involved in the breaking down of cell walls to increase the parasitism of oomycetes (Richards et al., 2011).

Even when an organism horizontally acquires a gene that leads to a selective advantage, the advantage might not have a short-term impact on the recipient’s ecology, and changes might only be significant when considered in an evolutionary timescale (Hiltunen et al., 2017). Similarly, the ecological benefits of an adaptation acquired by a sporadic HGT event could dissipate over time (Hiltunen et al., 2017). In these cases, the impacts to the ecosystem of a casual HGT event are difficult to assess, and risk assessments cannot rely on considering just HGT frequency, as this is not a good prognostic tool for long term effects of HGT (Pettersen et al., 2005).

Many adverse effects owing to the potential of HGT of the transgene from GM plants to other organisms, including humans, are gene dependent. These effects include their potential role in allergenicity, pathogenicity, virulence, toxicity and other environmental effects. Therefore, these potential effects should be evaluated on a case-by-case basis in the context of the proposed activities in the risk assessment of each GM plant if occurrence of HGT is considered more likely than when dealing with the non-GM plant.

For example, one of the most common concerns regarding GM plant safety is the potential environmental and health consequences of HGT with regards to prokaryotic-derived antibiotic resistance genes, which are predominantly used as markers to select for the transformation event. Should HGT and subsequent integration occur to the gut microflora of consumers, including humans, the concern relates to the proliferation of antibiotic resistant strains of harmful bacteria which would be harder to control (Rizzi et al., 2012). These concerns have been previously assessed (see EFSA (2017) and references within Woegerbauer et al. (2015)) and there is no account of such an event occurring from a GM plant source under field conditions (Demanèche et al., 2008; Pilate et al., 2016; EFSA et al., 2017; Tsatsakis et al., 2017; Hily et al., 2018). In addition, the presence of these resistance genes has not significantly increased antibiotic resistance in the clinical setting (Breyer et al., 2014). Furthermore, natural antibiotic and herbicide resistance genes are found in widely dispersed soil microorganisms, often on mobile genetic elements (Pontiroli et al., 2007) and the HGT of these naturally occurring genes has previously been described by Domingues et al. (2012). Therefore, these naturally occurring microorganisms, as well as the vast pool of available antibiotic resistance genes already naturally present in the intestinal microflora in the human GIT, are far more likely to be the HGT source for these resistance genes than GM plants (Pontiroli et al., 2007; Tothova et al., 2010; Huddleston, 2014).

That stated, the evaluation of antibiotic resistance genes in GM plants needs to be considered on a case-by-case basis and regulators at the European Food Safety Authority (EFSA) have adopted the classification of these genes into three risk groups. These groups class antibiotic resistance genes based on their abundance in the environment and their significance to human and veterinary medicine, reviewed in De Santis et al. (2018). Regulators in Europe have also issued a directive to phase out antibiotic resistance genes used in GM plants that have adverse effects on human health and the environment [Directive 2001/18/EC of the European Parliament and of the Council (on the deliberate release into the environment of genetically modified organisms)] (Garcia, 2006). As such, biotechnologists are encouraged to develop GM plants without the use of antibiotic resistance gene markers (Breyer et al., 2014). In Australia, the risk assessment process considers the background presence of the gene(s) used in the genetic modification “Antibiotic resistance marker genes commonly used in the selection process for generating GM plants are derived from soil bacteria abundant in the environment. Therefore, exposure to an antibiotic resistance gene, or to the protein encoded by such a gene, derived from a GMO, may or may not be significant against the naturally occurring background” (OGTR, 2013).

## 6 Conclusion

HGT is most prominent in prokaryotes, as many lack sexual recombination and thus employ HGT as a mechanism for adaptation to the environment. For example, the beneficial acquisition of antibiotic resistance through HGT in a clinical setting, and several other cases of HGT have been researched and documented in this context. Despite the significant role that HGT has played in the evolution of eukaryotic genomes, nuclear HGT events between multicellular eukaryotes are considered scarce, when compared to those between prokaryotes and in either direction between prokaryotes and eukaryotes (Richardson and Palmer, 2007; Keeling and Palmer, 2008; Keeling, 2009; Bock, 2010; Huang, 2013; Gao et al., 2014; Schönknecht et al., 2014). Specifically, scarcity of HGT between plants can be attributed to the need of a vector to facilitate the transfer (Mahelka et al., 2017).

Overall, the frequency of HGT for all organisms, including viruses and bacteria, is orders of magnitude lower than gene transfer by sexual or asexual reproduction. This is due to HGT needing to overcome numerous barriers, such as those related to the transfer, incorporation, and transmission of the DNA between organisms. In eukaryotes, additional barriers are needed to be overcome, where the DNA may first need to be transferred from the somatic to germ cell line and then be transferred to the recipient’s offspring.

The advances in whole genome sequencing and comparative genomics demonstrates that, although historical, HGT events in eukaryotic organisms may have previously been underestimated. (Bock, 2010; Crisp et al., 2015; Drezen et al., 2017; Quispe-Huamanquispe et al., 2017; Sieber et al., 2017; Matveeva and Otten, 2019). However, by the publication date of this manuscript, there have been no reports of adverse impacts on human health or environmental safety as a direct or indirect result of HGT from GM plants. Moreover, in the Australian context, GM plants approved for environmental release often contain genes and regulatory sequences that originate from naturally occurring organisms that are already present in the environment. Therefore, the potential for unintended adverse effects of HGT of the inserted genetic material is unlikely to be greater than those by its naturally occurring genetic counterparts. As such, the potential for adverse effects to human and animal health and to the environment as a result of HGT from GM plants already authorised for environmental release in Australia are highly unlikely. However, it is worth considering that, by exchanging the host of the introduced genetic material, a closer physical association to a potential recipient might be enabled, potentially increasing the likelihood for HGT. Furthermore, with recent advances in genome editing, non-food plants, such as *N. benthamiana*, are likely to be genetically modified with DNA sequences encoding components for production of pharmaceuticals and vaccines (Bally et al., 2018). The DNA sequences in such GM plants may be novel or synthetic, and therefore are unlikely to already be present in the environment. This would pose new challenges to gene technology regulators in conducting their risk analysis, as a direct comparator is not immediately apparent. The Australian approach provided by the Regulator’s Risk Analysis Framework (OGTR, 2013) would still allow the risk assessment of any novel or synthetic DNA sequence in the GM plant to be conducted. Similarly, the risks associated from gene-edited plants would also follow the above processes, noting that in Australia, organisms modified *via* Site Directed Nucleases (SDN) without guide RNAs (SDN-1) are organisms that are not genetically modified organisms under Schedule 1 of the Gene Technology Regulations 2001 for regulatory purposes following the changes made to the Australian Regulations in 2019 (OGTR, 2020; O’Sullivan et al., 2022).

## Footnotes

1 No application has been received for the environmental release of Agrisure^®^ Duracade™ 5222 corn in Australia by the publication date of this manuscript.

## References

[B1] ACRE (Advisory Committee on Releases to the Environment) (2013). Report 1: Towards an Evidence-Based Regulatory System for GMOs. London.

[B2] Acuña R. Padilla B. E. Flórez-Ramos C. P. Rubio J. D. Herrera J. C. Benavides P. (2012). Adaptive Horizontal Transfer of a Bacterial Gene to an Invasive Insect Pest of Coffee. Proc. Natl. Acad. Sci. U. S. A. 109 (11), 4197–4202. 10.1073/pnas.1121190109 22371593 PMC3306691

[B3] Anderson M. T. Seifert H. S. (2011). Opportunity and Means: Horizontal Gene Transfer from the Human Host to a Bacterial Pathogen. mBio 2 (1), e00005–00011. 10.1128/mBio.00005-11 21325040 PMC3042738

[B4] Andersson J. O. (2005). Lateral Gene Transfer in Eukaryotes. CMLS. Cell. Mol. Life Sci. 62 (11), 1182–1197. 10.1007/s00018-005-4539-z 15761667 PMC11138376

[B5] Badosa E. Moreno C. Montesinos E. (2004). Lack of Detection of Ampicillin Resistance Gene Transfer from Bt176 Transgenic Corn to Culturable Bacteria under Field Conditions. FEMS Microbiol. Ecol. 48 (2), 169–178. 10.1016/j.femsec.2004.01.005 19712400

[B6] Bally J. Jung H. Mortimer C. Naim F. Philips J. G. Hellens R. (2018). The Rise and Rise of *Nicotiana Benthamiana*: A Plant for All Reasons. Annu. Rev. Phytopathol. 56 (1), 405–426. 10.1146/annurev-phyto-080417-050141 30149789

[B7] Beever D. E. Kemp C. F. (2000). Safety Issues Associated with the DNA in Animal Feed Derived from Genetically Modified Crops. A Review of Scientific and Regulatory Procedures. Nutr. Abstr. Rev. Ser. B, Livest. Feed Feed. 70 (3), 197–204.

[B8] Beever D. E. Phipps R. H. (2001). The Fate of Plant DNA and Novel Proteins in Feeds for Farm Livestock: A United Kingdom Perspective. J. Animal Sci. 79, E290–E295. 10.2527/jas2001.79E-SupplE290x

[B9] Bertolla F. Simonet P. (1999). Horizontal Gene Transfers in the Environment: Natural Transformation as a Putative Process for Gene Transfers between Transgenic Plants and Microorganisms. Res. Microbiol. 150 (6), 375–384. 10.1016/S0923-2508(99)80072-2 10466405

[B10] Biosafety Clearing-House (2019). Living Modified Organism (LMO) Registry. Available: http://bch.cbd.int/database/lmo-registry/(Accessed 19 11, 2019).

[B11] Blokesch M. (2016). Natural Competence for Transformation. Curr. Biol. 26 (21), R1126–R1130. 10.1016/j.cub.2016.08.058 27825443

[B12] Bock R. (2010). The Give-And-Take of DNA: Horizontal Gene Transfer in Plants. Trends Plant Sci. 15 (1), 11–22. 10.1016/j.tplants.2009.10.001 19910236

[B13] Boothby T. C. Goldstein B. (2016). Reply to Bemm et al. and Arakawa: Identifying foreign genes in independent *Hypsibius dujardini* genome assemblies. Proc. Natl. Acad. Sci. U. S. A. 113 (22), E3058–E3061. 10.1073/pnas.1601149113 27173900 PMC4896697

[B14] Boto L. (2010). Horizontal Gene Transfer in Evolution: Facts and Challenges. Proc. R. Soc. B 277 (1683), 819–827. 10.1098/rspb.2009.1679 PMC2842723 19864285

[B15] Bray C. M. West C. E. (2005). DNA Repair Mechanisms in Plants: Crucial Sensors and Effectors for the Maintenance of Genome Integrity. New Phytol. 168 (3), 511–528. 10.1111/j.1469-8137.2005.01548.x 16313635

[B16] Breyer D. Kopertekh L. Reheul D. (2014). Alternatives to Antibiotic Resistance Marker Genes for *In Vitro* Selection of Genetically Modified Plants – Scientific Developments, Current Use, Operational Access and Biosafety Considerations. Crit. Rev. Plant Sci. 33 (4), 286–330. 10.1080/07352689.2013.870422

[B17] Brigulla M. Wackernagel W. (2010). Molecular Aspects of Gene Transfer and Foreign DNA Acquisition in Prokaryotes with Regard to Safety Issues. Appl. Microbiol. Biotechnol. 86 (4), 1027–1041. 10.1007/s00253-010-2489-3 20191269

[B18] Broaders E. Gahan C. G. Marchesi J. R. (2013). Mobile Genetic Elements of the Human Gastrointestinal Tract: Potential for Spread of Antibiotic Resistance Genes. Gut Microbes 4 (4), 271–280. 10.4161/gmic.24627 23651955 PMC3744512

[B19] Calsamiglia S. Hernandez B. Hartnell G. F. Phipps R. (2007). Effects of Corn Silage Derived from a Genetically Modified Variety Containing Two Transgenes on Feed Intake, Milk Production, and Composition, and the Absence of Detectable Transgenic Deoxyribonucleic Acid in Milk in Holstein Dairy Cows. J. Dairy Sci. 90 (10), 4718–4723. 10.3168/jds.2007-0286 17881694

[B20] Catoni M. Noris E. Vaira A. M. Jonesman T. Matić S. Soleimani R. (2018). Virus-mediated Export of Chromosomal DNA in Plants. Nat. Commun. 9 (1), 5308. 10.1038/s41467-018-07775-w 30546019 PMC6293997

[B21] Chen K. Otten L. (2017). Natural *Agrobacterium* Transformants: Recent Results and Some Theoretical Considerations. Front. Plant Sci. 8 (1600). 10.3389/fpls.2017.01600 PMC5606197 28966626

[B22] Christin P.-A. Edwards Erika J. Besnard G. Boxall Susanna F. Gregory R. Kellogg Elizabeth A. (2012). Adaptive Evolution of C_4_ Photosynthesis through Recurrent Lateral Gene Transfer. Curr. Biol. 22 (5), 445–449. 10.1016/j.cub.2012.01.054 22342748

[B23] Crisp A. Boschetti C. Perry M. Tunnacliffe A. Micklem G. (2015). Expression of Multiple Horizontally Acquired Genes Is a Hallmark of Both Vertebrate and Invertebrate Genomes. Genome Biol. 16 (1), 50. 10.1186/s13059-015-0607-3 25785303 PMC4358723

[B24] Danchin E. G. J. Rosso M.-N. Vieira P. de Almeida-Engler J. Coutinho P. M. Henrissat B. (2010). Multiple Lateral Gene Transfers and Duplications Have Promoted Plant Parasitism Ability in Nematodes. Proc. Natl. Acad. Sci. U. S. A. 107 (41), 17651–17656. 10.1073/pnas.1008486107 20876108 PMC2955110

[B25] Davis C. C. Xi Z. (2015). Horizontal Gene Transfer in Parasitic Plants. Curr. Opin. Plant Biol. 26, 14–19. 10.1016/j.pbi.2015.05.008 26051213

[B26] de la Casa-Esperón E. (2012). Horizontal Transfer and the Evolution of Host-Pathogen Interactions. Int. J. Evol. Biol. 9, 1. 10.1155/2012/679045 PMC3513734 23227424

[B27] De Santis B. Stockhofe N. Wal J.-M. Weesendorp E. Lallès J.-P. van Dijk J. (2018). Case Studies on Genetically Modified Organisms (GMOs): Potential Risk Scenarios and Associated Health Indicators. Food Chem. Toxicol. 117, 36–65. 10.1016/j.fct.2017.08.033 28859885

[B28] de Vries J. Meier P. Wackernagel W. (2001). The Natural Transformation of the Soil Bacteria *Pseudomonas Stutzeri* and *Acinetobacter* Sp. By Transgenic Plant DNA Strictly Depends on Homologous Sequences in the Recipient Cells. FEMS Microbiol. Lett. 195 (2), 211–215. 10.1111/j.1574-6968.2001.tb10523.x 11179654

[B29] Deaville E. R. Maddison B. C. (2005). Detection of Transgenic and Endogenous Plant DNA Fragments in the Blood, Tissues, and Digesta of Broilers. J. Agric. Food Chem. 53 (26), 10268–10275. 10.1021/jf051652f 16366726

[B30] Demanèche S. Monier J.-M. Dugat-Bony E. Simonet P. (2011). Exploration of Horizontal Gene Transfer between Transplastomic Tobacco and Plant-Associated Bacteria. FEMS Microbiol. Ecol. 78 (1), 129–136. 10.1111/j.1574-6941.2011.01126.x 21564143

[B31] Demanèche S. Sanguin H. Poté J. Navarro E. Bernillon D. Mavingui P. (2008). Antibiotic-resistant Soil Bacteria in Transgenic Plant Fields. Proc. Natl. Acad. Sci. U. S. A. 105 (10), 3957–3962. 10.1073/pnas.0800072105 18292221 PMC2268783

[B32] Deni J. Message B. Chioccioli M. Tepfer D. (2005). Unsuccessful Search for DNA Transfer from Transgenic Plants to Bacteria in the Intestine of the Tobacco Horn Worm, *Manduca Sexta* . Transgenic Res. 14 (2), 207–215. 10.1007/s11248-004-6701-z 16022391

[B33] Domingues S. Nielsen K. M. da Silva G. J. (2012). Various Pathways Leading to the Acquisition of Antibiotic Resistance by Natural Transformation. Mob. Genet. Elem. 2 (6), 257–260. 10.4161/mge.23089 PMC3575418 23482877

[B34] Drezen J.-M. Josse T. Bézier A. Gauthier J. Huguet E. Herniou E. A. (2017). Impact of Lateral Transfers on the Genomes of Lepidoptera. Genes. 8 (11), 315. 10.3390/genes8110315 PMC5704228 29120392

[B35] Dröge M. Pühler A. Selbitschka W. (1998). Horizontal Gene Transfer as a Biosafety Issue: A Natural Phenomenon of Public Concern. J. Biotechnol. 64 (1), 75–90. 10.1016/S0168-1656(98)00105-9 9823660

[B36] Dunning Hotopp J. C. Clark M. E. Oliveira D. C. Foster J. M. Fischer P. Munoz Torres M. C. (2007). Widespread Lateral Gene Transfer from Intracellular Bacteria to Multicellular Eukaryotes. Science 317 (5845), 1753–1756. 10.1126/science.1142490 17761848

[B37] Dunning L. T. Olofsson J. K. Parisod C. Choudhury R. R. Moreno-Villena J. J. Yang Y. (2019). Lateral Transfers of Large DNA Fragments Spread Functional Genes Among Grasses. Proc. Natl. Acad. Sci. U. S. A. 116 (10), 4416–4425. 10.1073/pnas.1810031116 30787193 PMC6410850

[B38] Dupont P.-Y. Cox M. P. (2017). Genomic Data Quality Impacts Automated Detection of Lateral Gene Transfer in Fungi. G3 Genes.|Genomes|Genetics 7 (4), 1301–1314. 10.1534/g3.116.038448 28235827 PMC5386878

[B39] EFSA Gennaro A. Gomes A. Herman L. Nogue F. Papadopoulou N. (2017). Explanatory note on DNA sequence similarity searches in the context of the assessment of horizontal gene transfer from plants to microorganisms. EFSA Support. Publ. 14 (7), 1273E. 10.2903/sp.efsa.2017.EN-1273

[B40] Fournier G. P. Andam C. P. Gogarten J. P. (2015). Ancient Horizontal Gene Transfer and the Last Common Ancestors. BMC Evol. Biol. 15 (1), 70. 10.1186/s12862-015-0350-0 25897759 PMC4427996

[B41] Fuentes I. Stegemann S. Golczyk H. Karcher D. Bock R. (2014). Horizontal Genome Transfer as an Asexual Path to the Formation of New Species. Nature 511 (7508), 232–235. 10.1038/nature13291 24909992

[B42] Gao C. Ren X. Mason A. S. Liu H. Xiao M. Li J. (2014). Horizontal Gene Transfer in Plants. Funct. Integr. Genomics 14 (1), 23–29. 10.1007/s10142-013-0345-0 24132513

[B43] Garcia P. R. (2006). Directive 2001/18/EC on the Deliberate Release into the Environment of GMOs: An Overview and the Main Provisions for Placing on the Market. J. Eur. Environ. Plan. Law 3 (1), 3–12. 10.1163/187601006X00029

[B44] Gardner C. M. Volkoff S. J. Gunsch C. K. (2019). Examining the Behavior of Crop-Derived Antibiotic Resistance Genes in Anaerobic Sludge Batch Reactors under Thermophilic Conditions. Biotechnol. Bioeng. 116 (11), 3063–3071. 10.1002/bit.27134 31388983

[B45] Garnatje T. Garcia S. Vilatersana R. Vallès J. (2006). Genome Size Variation in the Genus *Carthamus* (Asteraceae, Cardueae): Systematic Implications and Additive Changes during Allopolyploidization. Ann. Bot. 97 (3), 461–467. 10.1093/aob/mcj050 16390843 PMC2803645

[B46] Gebhard F. Smalla K. (1999). Monitoring Field Releases of Genetically Modified Sugar Beets for Persistence of Transgenic Plant DNA and Horizontal Gene Transfer. FEMS Microbiol. Ecol. 28 (3), 261–272. 10.1111/j.1574-6941.1999.tb00581.x

[B47] Gelvin S. B. (2003). *Agrobacterium*-mediated Plant Transformation: the Biology behind the “Gene-jockeying” Tool. Microbiol. Mol. Biol. Rev. 67 (1), 16–37. 10.1128/mmbr.67.1.16-37.2003 12626681 PMC150518

[B48] Gelvin S. B. (2017). Integration of *Agrobacterium* T-DNA into the Plant Genome. Annu. Rev. Genet. 51 (1), 195–217. 10.1146/annurev-genet-120215-035320 28853920

[B49] EFSA Gennaro A. Gomes A. Herman L. Nogue F. Papadopoulou N. (2017). Explanatory Note on DNA Sequence Similarity Searches in the Context of the Assessment of Horizontal Gene Transfer from Plants to Microorganisms. EFSA Support. Publ. 14 (7), 1273E. 10.2903/sp.efsa.2017.EN-1273

[B50] Ghoshal K. Theilmann J. Reade R. Maghodia A. Rochon D. A. (2015). Encapsidation of Host RNAs by *Cucumber Necrosis Virus* Coat Protein during Both Agroinfiltration and Infection. J. Virol. 89 (21), 10748–10761. 10.1128/jvi.01466-15 26269190 PMC4621115

[B51] Gladyshev E. A. Meselson M. Arkhipova I. R. (2008). Massive Horizontal Gene Transfer in Bdelloid Rotifers. Science 320 (5880), 1210–1213. 10.1126/science.1156407 18511688

[B52] Graham L. A. Lougheed S. C. Ewart K. V. Davies P. L. (2008). Lateral Transfer of a Lectin-like Antifreeze Protein Gene in Fishes. PLOS ONE 3 (7), e2616. 10.1371/journal.pone.0002616 18612417 PMC2440524

[B53] Grbic M. Van Leeuwen T. Clark R. M. Rombauts S. Rouze P. Grbic V. (2011). The Genome of *Tetranychus Urticae* Reveals Herbivorous Pest Adaptations. Nature 479 (7374), 487–492. 10.1038/nature10640 22113690 PMC4856440

[B54] Gurdon C. Svab Z. Feng Y. Kumar D. Maliga P. (2016). Cell-to-cell Movement of Mitochondria in Plants. Proc. Natl. Acad. Sci. U. S. A. 113 (12), 3395–3400. 10.1073/pnas.1518644113 26951647 PMC4812711

[B55] Haegeman A. Jones J. T. Danchin E. G. J. (2011). Horizontal Gene Transfer in Nematodes: A Catalyst for Plant Parasitism? Mol. Plant-Microbe Interact. 24 (8), 879–887. 10.1094/mpmi-03-11-0055 21539433

[B56] Hawes M. C. Curlango-Rivera G. Xiong Z. Kessler J. O. (2012). Roles of Root Border Cells in Plant Defense and Regulation of Rhizosphere Microbial Populations by Extracellular DNA ‘trapping. Plant Soil 355 (1), 1–16. 10.1007/s11104-012-1218-3

[B57] Hecht M. M. Nitz N. Araujo P. F. Sousa A. O. Rosa A. d. C. Gomes D. A. (2010). Inheritance of DNA Transferred from American Trypanosomes to Human Hosts. PLOS ONE 5 (2), e9181. 10.1371/journal.pone.0009181 20169193 PMC2820539

[B58] Heck G. R. CaJacob C. A. Padgette S. R. (2003). “Discovery, Development, and Commercialization of Roundup Ready® Crops,” in Plant Biotechnology 2002 and beyond: Proceedings of the 10th IAPTC&B Congress June 23–28, 2002 Orlando, Florida, U.S.A.I.K. Vasil. (Dordrecht: Springer Netherlands), 139–142.

[B59] Hendriksma H. P. Kuting M. Hartel S. Nather A. Dohrmann A. B. Steffan-Dewenter I. (2013). Effect of Stacked Insecticidal Cry Proteins from Maize Pollen on Nurse Bees (*Apis mellifera Carnica*) and Their Gut Bacteria. PLOS ONE 8 (3), e59589. 10.1371/journal.pone.0059589 23533634 PMC3606186

[B60] Hibdige S. G. S. Raimondeau P. Christin P.-A. Dunning L. T. (2021). Widespread Lateral Gene Transfer Among Grasses. New Phytol. 230 (6), 2474–2486. 10.1111/nph.17328 33887801

[B61] Hiltunen T. Virta M. Laine A.-L. (2017). Antibiotic Resistance in the Wild: an Eco-Evolutionary Perspective. Phil. Trans. R. Soc. B 372 (1712), 20160039. 10.1098/rstb.2016.0039 27920384 PMC5182435

[B62] Hily J.-M. Demanèche S. Poulicard N. Tannières M. Djennane S. Beuve M. (2018). Metagenomic-based Impact Study of Transgenic Grapevine Rootstock on its Associated Virome and Soil Bacteriome. Plant Biotechnol. J. 16 (1), 208–220. 10.1111/pbi.12761 28544449 PMC5785345

[B63] Hohlweg U. Doerfler W. (2001). On the Fate of Plant or Other Foreign Genes upon the Uptake in Food or after Intramuscular Injection in Mice. Mol. Gen. Genomics 265 (2), 225–233. 10.1007/s004380100450 11361332

[B64] Huang J. (2013). Horizontal Gene Transfer in Eukaryotes: the Weak-Link Model. BioEssays 35 (10), 868–875. 10.1002/bies.201300007 24037739 PMC4033532

[B65] Huddleston J. R. (2014). Horizontal Gene Transfer in the Human Gastrointestinal Tract: Potential Spread of Antibiotic Resistance Genes. Infect. Drug Resist. 7, 167–176. 10.2147/IDR.S48820 25018641 PMC4073975

[B66] Hülter N. Wackernagel W. (2008). Double Illegitimate Recombination Events Integrate DNA Segments through Two Different Mechanisms during Natural Transformation of *Acinetobacter Baylyi* . Mol. Microbiol. 67 (5), 984–995. 10.1111/j.1365-2958.2007.06096.x 18194157

[B67] Hultman J. Tamminen M. Pärnänen K. Cairns J. Karkman A. Virta M. (2018). Host Range of Antibiotic Resistance Genes in Wastewater Treatment Plant Influent and Effluent. FEMS Microbiol. Ecol. 94 (4). 10.1093/femsec/fiy038 PMC5939699 29514229

[B68] Jennings J. C. Kolwyck D. C. Kays S. B. Whetsell A. J. Surber J. B. Cromwell G. L. (2003). Determining whether Transgenic and Endogenous Plant DNA and Transgenic Protein Are Detectable in Muscle from Swine Fed Roundup Ready Soybean Meal1, 2, 3. J. Animal Sci. 81 (6), 1447–1455. 10.2527/2003.8161447x 12817492

[B69] Jonas D. A. Elmadfa I. Engel K. H. Heller K. J. Kozianowski G. König A. (2001). Safety Considerations of DNA in Food. Ann. Nutr. Metab. 45 (6), 235–254. 10.1159/000046734 11786646

[B70] Kay E. Vogel T. M. Bertolla F. Nalin R. Simonet P. (2002). *In Situ* transfer of Antibiotic Resistance Genes from Transgenic (Transplastomic) Tobacco Plants to Bacteria. Appl. Environ. Microbiol. 68 (7), 3345–3351. 10.1128/aem.68.7.3345-3351.2002 12089013 PMC126776

[B71] Keeling P. J. (2009). Functional and Ecological Impacts of Horizontal Gene Transfer in Eukaryotes. Curr. Opin. Genet. Dev. 19 (6), 613–619. 10.1016/j.gde.2009.10.001 19897356

[B72] Keeling P. J. Palmer J. D. (2008). Horizontal Gene Transfer in Eukaryotic Evolution. Nat. Rev. Genet. 9 (8), 605–618. 10.1038/nrg2386 18591983

[B73] Keese P. (2008). Risks from GMOs Due to Horizontal Gene Transfer. Environ. Biosaf. Res. 7 (3), 123–149. 10.1051/ebr:2008014 18801324

[B74] Korwin-Kossakowska A. Sartowska K. Tomczyk G. Prusak B. Sender G. (2016). Health Status and Potential Uptake of Transgenic DNA by Japanese Quail Fed Diets Containing Genetically Modified Plant Ingredients over 10 Generations. Br. Poult. Sci. 57 (3), 415–423. 10.1080/00071668.2016.1162281 27095142

[B75] Kyndt T. Quispe D. Zhai H. Jarret R. Ghislain M. Liu Q. (2015). The Genome of Cultivated Sweet Potato Contains *Agrobacterium* T-DNAs with Expressed Genes: An Example of a Naturally Transgenic Food Crop. Proc. Natl. Acad. Sci. U. S. A. 112 (18), 5844–5849. 10.1073/pnas.1419685112 25902487 PMC4426443

[B76] Lang A. S. Zhaxybayeva O. Beatty J. T. (2012). Gene Transfer Agents: Phage-like Elements of Genetic Exchange. Nat. Rev. Microbiol. 10 (7), 472–482. 10.1038/nrmicro2802 22683880 PMC3626599

[B77] Latham J. R. Love M. Hilbeck A. (2017). The Distinct Properties of Natural and GM Cry Insecticidal Proteins. Biotechnol. Genet. Eng. Rev. 33 (1), 62–96. 10.1080/02648725.2017.1357295 28901209

[B78] Lerner A. Matthias T. Aminov R. (2017). Potential Effects of Horizontal Gene Exchange in the Human Gut. Front. Immunol. 8 (1630). 10.3389/fimmu.2017.01630 PMC5711824 29230215

[B79] Li F.-W. Villarreal J. C. Kelly S. Rothfels C. J. Melkonian M. Frangedakis E. (2014). Horizontal Transfer of an Adaptive Chimeric Photoreceptor from Bryophytes to Ferns. Proc. Natl. Acad. Sci. U. S. A. 111 (18), 6672–6677. 10.1073/pnas.1319929111 24733898 PMC4020063

[B80] Li M. Zhao J. Tang N. Sun H. Huang J. (2018). Horizontal Gene Transfer from Bacteria and Plants to the Arbuscular Mycorrhizal Fungus *Rhizophagus Irregularis* . Front. Plant Sci. 9 (701). 10.3389/fpls.2018.00701 PMC5982333 29887874

[B81] Lin T. Chen Z. Usha R. Stauffacher C. V. Dai J.-B. Schmidt T. (1999). The Refined Crystal Structure of Cowpea Mosaic Virus at 2.8 Å Resolution. Virology 265 (1), 20–34. 10.1006/viro.1999.0038 10603314

[B82] Losey J. E. Harmon J. Ballantyne F. Brown C. (1997). A Polymorphism Maintained by Opposite Patterns of Parasitism and Predation. Nature 388 (6639), 269–272. 10.1038/40849

[B83] Ma B. L. Blackshaw R. E. Roy J. He T. (2011). Investigation on Gene Transfer from Genetically Modified Corn (*Zea mays* L.) Plants to Soil Bacteria. J. Environ. Sci. Health, Part B 46 (7), 590–599. 10.1080/03601234.2011.586598 21722080

[B84] Mahelka V. Krak K. Kopecký D. Fehrer J. Šafář J. Bartoš J. (2017). Multiple Horizontal Transfers of Nuclear Ribosomal Genes between Phylogenetically Distinct Grass Lineages. Proc. Natl. Acad. Sci. U. S. A. 114 (7), 1726–1731. 10.1073/pnas.1613375114 28137844 PMC5320982

[B85] Maliga P. Bock R. (2011). Plastid Biotechnology: Food, Fuel, and Medicine for the 21st Century. Plant Physiol. 155 (4), 1501–1510. 10.1104/pp.110.170969 21239622 PMC3091108

[B86] Mao J. Lu T. (2016). Population-dynamic Modeling of Bacterial Horizontal Gene Transfer by Natural Transformation. Biophysical J. 110 (1), 258–268. 10.1016/j.bpj.2015.11.033 PMC4806214 26745428

[B87] Matveeva T. V. Otten L. (2019). Widespread Occurrence of Natural Genetic Transformation of Plants by *Agrobacterium* . Plant Mol. Biol. 101, 415–437. 10.1007/s11103-019-00913-y 31542868

[B88] Mazza R. Soave M. Morlacchini M. Piva G. Marocco A. (2005). Assessing the Transfer of Genetically Modified DNA from Feed to Animal Tissues. Transgenic Res. 14 (5), 775–784. 10.1007/s11248-005-0009-5 16245168

[B89] McAdams H. H. Srinivasan B. Arkin A. P. (2004). The Evolution of Genetic Regulatory Systems in Bacteria. Nat. Rev. Genet. 5 (3), 169–178. 10.1038/nrg1292 14970819

[B90] McDonald M. C. Taranto A. P. Hill E. Schwessinger B. Liu Z. Simpfendorfer S. (2019). Transposon-mediated Horizontal Transfer of the Host-specific Virulence Protein ToxA between Three Fungal Wheat Pathogens. mBio 10 (5), e01515–01519. 10.1128/mBio.01515-19 31506307 PMC6737239

[B91] Mitreva M. Smant G. Helder J. (2009). “Role of Horizontal Gene Transfer in the Evolution of Plant Parasitism Among Nematodes,” in Horizontal Gene Transfer: Genomes in Flux. Editors Gogarten M. B. Gogarten J. P. Olendzenski L. C. (Totowa, NJ: Humana Press), 517–535. 10.1007/978-1-60327-853-9_30 19271205

[B92] Miyashita H. Kuroki Y. Kretsinger R. H. Matsushima N. (2013). Horizontal Gene Transfer of Plant-specific Leucine-Rich Repeats between Plants and Bacteria. Nat. Sci. 5 (5), 580–598. 10.4236/ns.2013.55074

[B93] Mohr K. I. Tebbe C. C. (2007). Field Study Results on the Probability and Risk of a Horizontal Gene Transfer from Transgenic Herbicide-Resistant Oilseed Rape Pollen to Gut Bacteria of Bees. Appl. Microbiol. Biotechnol. 75 (3), 573–582. 10.1007/s00253-007-0846-7 17273854

[B94] Monier J.-M. Bernillon D. Kay E. Faugier A. Rybalka O. Dessaux Y. (2007). Detection of Potential Transgenic Plant DNA Recipients Among Soil Bacteria. Environ. Biosaf. Res. 6 (1-2), 71–83. 10.1051/ebr:2007036 17961481

[B95] Moran N. A. Jarvik T. (2010). Lateral Transfer of Genes from Fungi Underlies Carotenoid Production in Aphids. Science 328 (5978), 624–627. 10.1126/science.1187113 20431015

[B96] Nadal A. De Giacomo M. Einspanier R. Kleter G. Kok E. McFarland S. (2018). Exposure of Livestock to GM Feeds: Detectability and Measurement. Food Chem. Toxicol. 117, 13–35. 10.1016/j.fct.2017.08.032 28847764

[B97] Nawaz M. A. Mesnage R. Tsatsakis A. M. Golokhvast K. S. Yang S. H. Antoniou M. N. (2019). Addressing Concerns over the Fate of DNA Derived from Genetically Modified Food in the Human Body: A Review. Food Chem. Toxicol. 124, 423–430. 10.1016/j.fct.2018.12.030 30580028

[B98] Nemeth A. Wurz A. Artim L. Charlton S. Dana G. Glenn K. (2004). Sensitive PCR Analysis of Animal Tissue Samples for Fragments of Endogenous and Transgenic Plant DNA. J. Agric. Food Chem. 52 (20), 6129–6135. 10.1021/jf049567f 15453677

[B99] Netherwood T. Martín-Orúe S. M. O'Donnell A. G. Gockling S. Graham J. Mathers J. C. (2004). Assessing the Survival of Transgenic Plant DNA in the Human Gastrointestinal Tract. Nat. Biotechnol. 22, 204–209. 10.1038/nbt934 14730317

[B100] Nicolia A. Manzo A. Veronesi F. Rosellini D. (2014). An Overview of the Last 10 Years of Genetically Engineered Crop Safety Research. Crit. Rev. Biotechnol. 34 (1), 77–88. 10.3109/07388551.2013.823595 24041244

[B101] Nielsen K. M. Bones A. M. Smalla K. van Elsas J. D. (1998). Horizontal Gene Transfer from Transgenic Plants to Terrestrial Bacteria – a Rare Event? FEMS Microbiol. Rev. 22 (2), 79–103. 10.1016/s0168-6445(98)00009-6 9729765

[B102] Nikoh N. Nakabachi A. (2009). Aphids Acquired Symbiotic Genes *via* Lateral Gene Transfer. BMC Biol. 7, 12. 10.1186/1741-7007-7-12 19284544 PMC2662799

[B103] Nikoh N. Tanaka K. Shibata F. Kondo N. Hizume M. Shimada M. (2008). *Wolbachia* Genome Integrated in an Insect Chromosome: Evolution and Fate of Laterally Transferred Endosymbiont Genes. Genome Res. 18 (2), 272–280. 10.1101/gr.7144908 18073380 PMC2203625

[B104] Nikolaidis N. Doran N. Cosgrove D. J. (2013). Plant Expansins in Bacteria and Fungi: Evolution by Horizontal Gene Transfer and Independent Domain Fusion. Mol. Biol. Evol. 31 (2), 376–386. 10.1093/molbev/mst206 24150040

[B105] Niu L. Ma W. Lei C. Jurat-Fuentes J. L. Chen L. (2017). Herbicide and Insect Resistant Bt Cotton Pollen Assessment Finds No Detrimental Effects on Adult Honey Bees. Environ. Pollut. 230, 479–485. 10.1016/j.envpol.2017.06.094 28688300

[B106] Noon J. B. Baum T. J. (2016). Horizontal Gene Transfer of Acetyltransferases, Invertases and Chorismate Mutases from Different Bacteria to Diverse Recipients. BMC Evol. Biol. 16, 74. 10.1186/s12862-016-0651-y 27068610 PMC4828791

[B107] Nordgård L. Brusetti L. Raddadi N. Traavik T. Averhoff B. Nielsen K. M. (2012). An Investigation of Horizontal Transfer of Feed Introduced DNA to the Aerobic Microbiota of the Gastrointestinal Tract of Rats. BMC Res. Notes 5 (1), 170. 10.1186/1756-0500-5-170 22463741 PMC3364145

[B108] OGTR (2020). Gene Technology Regulations 2001. Canberra: Office of Parliamentary Counsel.

[B109] OGTR (2013). Risk Analysis Framework 2013. Canberra: Office of the Gene Technology Regulator.

[B110] O’Sullivan G. M. Philips J. G. Mitchell H. J. Dornbusch M. Rasko J. E. J. (2022). 20 Years of Legislation - How Australia Has Responded to the Challenge of Regulating Genetically Modified Organisms in the Clinic. Front. Med. 9, 883434. 10.3389/fmed.2022.883434 PMC9127347 35620726

[B111] Parrott W. Chassy B. Ligon J. Meyer L. Petrick J. Zhou J. (2010). Application of Food and Feed Safety Assessment Principles to Evaluate Transgenic Approaches to Gene Modulation in Crops. Food Chem. Toxicol. 48 (7), 1773–1790. 10.1016/j.fct.2010.04.017 20399824

[B112] Pettersen A. K. Bohn T. Primicerio R. Shorten P. R. Soboleva T. K. Nielsen K. M. (2005). Modeling Suggests Frequency Estimates Are Not Informative for Predicting the Long-Term Effect of Horizontal Gene Transfer in Bacteria. Environ. Biosaf. Res. 4 (4), 223–233. 10.1051/ebr:2006008 16827550

[B113] Philips J. G. Naim F. Lorenc M. T. Dudley K. J. Hellens R. P. Waterhouse P. M. (2017). The Widely Used *Nicotiana Benthamiana* 16c Line Has an Unusual T-DNA Integration Pattern Including a Transposon Sequence. PLOS ONE 12 (2), e0171311. 10.1371/journal.pone.0171311 28231340 PMC5322946

[B114] Pilate G. Allona I. Boerjan W. Déjardin A. Fladung M. Gallardo F. (2016). “Lessons from 25 Years of GM Tree Field Trials in Europe and Prospects for the Future,” in Biosafety of Forest Transgenic Trees: Improving the Scientific Basis for Safe Tree Development and Implementation of EU Policy Directives. Editors Vettori C. Gallardo F. Häggman H. Kazana V. Migliacci F. Pilate G. (Dordrecht: Springer Netherlands), 67–100.

[B115] Pontiroli A. Ceccherini M.-T. Poté J. Wildi W. Kay E. Nannipieri P. (2010). Long-term Persistence and Bacterial Transformation Potential of Transplastomic Plant DNA in Soil. Res. Microbiol. 161 (5), 326–334. 10.1016/j.resmic.2010.04.009 20493252

[B116] Pontiroli A. Rizzi A. Simonet P. Daffonchio D. Vogel T. M. Monier J.-M. (2009). Visual Evidence of Horizontal Gene Transfer between Plants and Bacteria in the Phytosphere of Transplastomic Tobacco. Appl. Environ. Microbiol. 75 (10), 3314–3322. 10.1128/AEM.02632-08 19329660 PMC2681637

[B117] Pontiroli A. Simonet P. Frostegard A. Vogel T. M. Monier J.-M. (2007). Fate of Transgenic Plant DNA in the Environment. Environ. Biosaf. Res. 6 (1-2), 15–35. 10.1051/ebr:2007037 17961478

[B118] Poté J. Ackermann R. Wildi W. (2009). Plant Leaf Mass Loss and DNA Release in Freshwater Sediments. Ecotoxicol. Environ. Saf. 72 (5), 1378–1383. 10.1016/j.ecoenv.2009.04.010 19419763

[B119] Prentice H. C. Li Y. Lönn M. Tunlid A. Ghatnekar L. (2015). A Horizontally Transferred Nuclear Gene Is Associated with Microhabitat Variation in a Natural Plant Population. Proc. R. Soc. B 282 (1821), 20152453. 10.1098/rspb.2015.2453 PMC4707765 26674953

[B120] Qiu H. Cai G. Luo J. Bhattacharya D. Zhang N. (2016). Extensive Horizontal Gene Transfers between Plant Pathogenic Fungi. BMC Biol. 14 (1), 41. 10.1186/s12915-016-0264-3 27215567 PMC4876562

[B121] Quispe-Huamanquispe D. G. Gheysen G. Kreuze J. F. (2017). Horizontal Gene Transfer Contributes to Plant Evolution: The Case of *Agrobacterium* T-DNAs. Front. Plant Sci. 8, 2015. 10.3389/fpls.2017.02015 29225610 PMC5705623

[B122] Ravenhall M. Škunca N. Lassalle F. Dessimoz C. (2015). Inferring Horizontal Gene Transfer. PLoS Comput. Biol. 11 (5), e1004095. 10.1371/journal.pcbi.1004095 26020646 PMC4462595

[B123] Raz Y. Tannenbaum E. (2010). The Influence of Horizontal Gene Transfer on the Mean Fitness of Unicellular Populations in Static Environments. Genetics 185 (1), 327–337. 10.1534/genetics.109.113613 20194966 PMC2870967

[B124] Rice D. W. Alverson A. J. Richardson A. O. Young G. J. Sanchez-Puerta M. V. Munzinger J. (2013). Horizontal Transfer of Entire Genomes *via* Mitochondrial Fusion in the Angiosperm *Amborella* . Science 342 (6165), 1468–1473. 10.1126/science.1246275 24357311

[B125] Richards T. A. Soanes D. M. Foster P. G. Leonard G. Thornton C. R. Talbot N. J. (2009). Phylogenomic Analysis Demonstrates a Pattern of Rare and Ancient Horizontal Gene Transfer between Plants and Fungi. Plant Cell 21 (7), 1897–1911. 10.1105/tpc.109.065805 19584142 PMC2729602

[B126] Richards T. A. Soanes D. M. Jones M. D. Vasieva O. Leonard G. Paszkiewicz K. (2011). Horizontal Gene Transfer Facilitated the Evolution of Plant Parasitic Mechanisms in the Oomycetes. Proc. Natl. Acad. Sci. U. S. A. 108 (37), 15258–15263. 10.1073/pnas.1105100108 21878562 PMC3174590

[B127] Richardson A. O. Palmer J. D. (2007). Horizontal Gene Transfer in Plants. J. Exp. Bot. 58 (1), 1–9. 10.1093/jxb/erl148 17030541

[B128] Rigden J. E. Dry I. B. Krake L. R. Rezaian M. A. (1996). Plant Virus DNA Replication Processes in *Agrobacterium*: Insight into the Origins of Geminiviruses? Proc. Natl. Acad. Sci. U. S. A. 93 (19), 10280–10284. 10.1073/pnas.93.19.10280 8816791 PMC38375

[B129] Rizzi A. Raddadi N. Sorlini C. Nordgrd L. Nielsen K. M. Daffonchio D. (2012). The Stability and Degradation of Dietary DNA in the Gastrointestinal Tract of Mammals: Implications for Horizontal Gene Transfer and the Biosafety of GMOs. Crit. Rev. Food Sci. Nutr. 52 (2), 142–161. 10.1080/10408398.2010.499480 22059960

[B130] Rose A. S. Bradley A. R. Valasatava Y. Duarte J. M. Prlić A. Rose P. W. (2018). NGL Viewer: Web-Based Molecular Graphics for Large Complexes. Bioinformatics 34 (21), 3755–3758. 10.1093/bioinformatics/bty419 29850778 PMC6198858

[B131] Rossi F. Morlacchini M. Fusconi G. Pietri A. Mazza R. Piva G. (2005). Effect of Bt Corn on Broiler Growth Performance and Fate of Feed-Derived DNA in the Digestive Tract. Poult. Sci. 84 (7), 1022–1030. 10.1093/ps/84.7.1022 16050119

[B132] Rumpho M. E. Worful J. M. Lee J. Kannan K. Tyler M. S. Bhattacharya D. (2008). Horizontal Gene Transfer of the Algal Nuclear Gene *psbO* to the Photosynthetic Sea Slug *Elysia Chlorotica* . Proc. Natl. Acad. Sci. U. S. A. 105 (46), 17867–17871. 10.1073/pnas.0804968105 19004808 PMC2584685

[B133] Sakamoto W. Takami T. (2018). Chloroplast DNA Dynamics: Copy Number, Quality Control and Degradation. Plant Cell Physiology 59 (6), 1120–1127. 10.1093/pcp/pcy084 29860378

[B134] Sanchez-Puerta M. V. Edera A. Gandini C. L. Williams A. V. Howell K. A. Nevill P. G. (2019). Genome-scale Transfer of Mitochondrial DNA from Legume Hosts to the Holoparasite *Lophophytum Mirabile* (Balanophoraceae). Mol. Phylogenetics Evol. 132, 243–250. 10.1016/j.ympev.2018.12.006 30528080

[B135] Sand K. K. Jelavić S. (2018). Mineral Facilitated Horizontal Gene Transfer: A New Principle for Evolution of Life? Front. Microbiol. 9 (2217). 10.3389/fmicb.2018.02217 PMC6167411 30319562

[B136] Schneider C. L. (2017). “Bacteriophage-Mediated Horizontal Gene Transfer: Transduction,” in Bacteriophages: Biology, Technology, Therapy. Editors Harper D. Abedon S. Burrowes B. McConville M. (Cham: Springer International Publishing), 1–42.

[B137] Schönknecht G. Weber A. P. M. Lercher M. J. (2014). Horizontal Gene Acquisitions by Eukaryotes as Drivers of Adaptive Evolution. BioEssays 36 (1), 9–20. 10.1002/bies.201300095 24323918

[B138] Schreiber M. Stein N. Mascher M. (2018). Genomic Approaches for Studying Crop Evolution. Genome Biol. 19 (1), 140. 10.1186/s13059-018-1528-8 30241487 PMC6151037

[B139] Schubbert R. Hohlweg U. Renz D. Doerfler W. (1998). On the Fate of Orally Ingested Foreign DNA in Mice: Chromosomal Association and Placental Transmission to the Fetus. Mol. Gen. Genet. 259 (6), 569–576. 10.1007/s004380050850 9819049

[B140] Schubbert R. Renz D. Schmitz B. Doerfler W. (1997). Foreign (M13) DNA Ingested by Mice Reaches Peripheral Leukocytes, Spleen, and Liver *via* the Intestinal Wall Mucosa and Can Be Covalently Linked to Mouse DNA. Proc. Natl. Acad. Sci. U. S. A. 94 (3), 961–966. 10.1073/pnas.94.3.961 9023365 PMC19622

[B141] Sharma R. Damgaard D. Alexander T. W. Dugan M. E. Aalhus J. L. Stanford K. (2006). Detection of Transgenic and Endogenous Plant DNA in Digesta and Tissues of Sheep and Pigs Fed Roundup Ready Canola Meal. J. Agric. Food Chem. 54 (5), 1699–1709. 10.1021/jf052459o 16506822

[B142] Shen J. Zhang Y. Havey M. J. Shou W. (2019). Copy Numbers of Mitochondrial Genes Change during Melon Leaf Development and Are Lower Than the Numbers of Mitochondria. Hortic. Res. 6 (1), 95. 10.1038/s41438-019-0177-8 31645953 PMC6804604

[B143] Shinozuka H. Hettiarachchige I. K. Shinozuka M. Cogan N. O. I. Spangenberg G. C. Cocks B. G. (2017). Horizontal Transfer of a SS-1, 6-glucanase Gene from an Ancestral Species of Fungal Endophyte to a Cool-Season Grass Host. Sci. Rep. 7 (1), 9024. 10.1038/s41598-017-07886-2 28831055 PMC5567365

[B144] Sieber K. B. Bromley R. E. Dunning Hotopp J. C. (2017). Lateral Gene Transfer between Prokaryotes and Eukaryotes. Exp. Cell Res. 358 (2), 421–426. 10.1016/j.yexcr.2017.02.009 28189637 PMC5550378

[B145] Sieradzki Z. Mazur M. Kwiatek K. Świątkiewicz S. Świątkiewicz M. Koreleski J. (2013). Assessing the Possibility of Genetically Modified DNA Transfer from GM Feed to Broiler, Laying Hen, Pig and Calf Tissues. Pol. J. Veterinary Sci. 16 (3), 435–441. 10.2478/pjvs-2013-0061 24195276

[B146] Sinn B. T. Barrett C. F. (2019). Ancient Mitochondrial Gene Transfer between Fungi and the Orchids. Mol. Biol. Evol. 37, 44–57. 10.1093/molbev/msz198 31504747

[B147] Soanes D. Richards T. A. (2014). Horizontal Gene Transfer in Eukaryotic Plant Pathogens. Annu. Rev. Phytopathol. 52, 583–614. 10.1146/annurev-phyto-102313-050127 25090479

[B148] Soucy S. M. Huang J. Gogarten J. P. (2015). Horizontal Gene Transfer: Building the Web of Life. Nat. Rev. Genet. 16 (8), 472–482. 10.1038/nrg3962 26184597

[B149] Stegemann S. Bock R. (2009). Exchange of Genetic Material between Cells in Plant Tissue Grafts. Science 324 (5927), 649–651. 10.1126/science.1170397 19407205

[B150] Stegemann S. Keuthe M. Greiner S. Bock R. (2012). Horizontal Transfer of Chloroplast Genomes between Plant Species. Proc. Natl. Acad. Sci. U. S. A. 109 (7), 2434–2438. 10.1073/pnas.1114076109 22308367 PMC3289295

[B151] Świątkiewicz M. Bednarek D. Markowski J. Hanczakowska E. Kwiate K. (2013). “Effect of Feeding Genetically Modified Maize and Soybean Meal to Sows on Their Reproductive Traits, Haematological Indices and Offspring Performance,” in Bulletin of the Veterinary Institute in Pulawy.

[B152] Thyssen G. Svab Z. Maliga P. (2012). Cell-to-cell Movement of Plastids in Plants. Proc. Natl. Acad. Sci. U. S. A. 109 (7), 2439–2443. 10.1073/pnas.1114297109 22308369 PMC3289365

[B154] Tothova T. Sobekova A. Holovska K. Legath J. Pristas P. Javorsky P. (2010). “Natural Glufosinate Resistance of Soil Microorganisms and GMO Safety,” in Open Life Sciences.

[B155] Tsatsakis A. M. Nawaz M. A. Kouretas D. Balias G. Savolainen K. Tutelyan V. A. (2017). Environmental Impacts of Genetically Modified Plants: A Review. Environ. Res. 156, 818–833. 10.1016/j.envres.2017.03.011 28347490

[B156] Ülker B. Li Y. Rosso M. G. Logemann E. Somssich I. E. Weisshaar B. (2008). T-DNA–mediated Transfer of *Agrobacterium Tumefaciens* Chromosomal DNA into Plants. Nat. Biotechnol. 26 (9), 1015–1017. 10.1038/nbt.1491 18758448

[B157] Van Eenennaam A. L. Young A. E. (2014). Prevalence and Impacts of Genetically Engineered Feedstuffs on Livestock Populations1. J. Animal Sci. 92 (10), 4255–4278. 10.2527/jas.2014-8124 25184846

[B158] van Elsas J. D. Turner S. Bailey M. J. (2003). Horizontal Gene Transfer in the Phytosphere. New Phytol. 157 (3), 525–537. 10.1046/j.1469-8137.2003.00697.x 33873398

[B159] Vogan A. A. Higgs P. G. (2011). The Advantages and Disadvantages of Horizontal Gene Transfer and the Emergence of the First Species. Biol. Direct 6, 1. 10.1186/1745-6150-6-1 21199581 PMC3043529

[B160] Walsh M. C. Buzoianu S. G. Gardiner G. E. Rea M. C. Gelencser E. Janosi A. (2011). Fate of Transgenic DNA from Orally Administered Bt Mon810 Maize and Effects on Immune Response and Growth in Pigs. PLOS ONE 6 (11), e27177. 10.1371/journal.pone.0027177 22132091 PMC3223173

[B161] Wickell D. A. Li F.-W. (2019). On the Evolutionary Significance of Horizontal Gene Transfers in Plants. New Phytol. 225, 113–117. 10.1111/nph.16022 31347197

[B162] Wilcks A. Jacobsen B. B. (2010). Lack of Detectable DNA Uptake by Transformation of Selected Recipients in Mono-Associated Rats. BMC Res. Notes 3 (1), 49. 10.1186/1756-0500-3-49 20193062 PMC2845597

[B163] Woegerbauer M. Zeinzinger J. Gottsberger R. A. Pascher K. Hufnagl P. Indra A. (2015). Antibiotic Resistance Marker Genes as Environmental Pollutants in GMO-Pristine Agricultural Soils in Austria. Environ. Pollut. 206, 342–351. 10.1016/j.envpol.2015.07.028 26232739

[B164] Wybouw N. Pauchet Y. Heckel D. G. Van Leeuwen T. (2016). Horizontal Gene Transfer Contributes to the Evolution of Arthropod Herbivory. Genome Biol. Evol. 8 (6), 1785–1801. 10.1093/gbe/evw119 27307274 PMC4943190

[B165] Wybouw N. Van Leeuwen T. Dermauw W. (2018). A Massive Incorporation of Microbial Genes into the Genome of *Tetranychus Urticae*, a Polyphagous Arthropod Herbivore. Insect Mol. Biol. 27 (3), 333–351. 10.1111/imb.12374 29377385

[B166] Xi Z. Wang Y. Bradley R. K. Sugumaran M. Marx C. J. Rest J. S. (2013). Massive Mitochondrial Gene Transfer in a Parasitic Flowering Plant Clade. PLoS Genet. 9 (2), e1003265. 10.1371/journal.pgen.1003265 23459037 PMC3573108

[B167] Xia J. Guo Z. Yang Z. Han H. Wang S. Xu H. (2021). Whitefly Hijacks a Plant Detoxification Gene that Neutralizes Plant Toxins. Cell 184 (7), 1693–1705.e17. 10.1016/j.cell.2021.02.014 33770502

[B168] Yang T. Chen Y. Wang X.-X. Dai C.-C. (2013). Plant Symbionts: Keys to the Phytosphere. Symbiosis 59 (1), 1–14. 10.1007/s13199-012-0190-2

[B169] Yang Z. Wafula E. K. Kim G. Shahid S. McNeal J. R. Ralph P. E. (2019). Convergent Horizontal Gene Transfer and Cross-Talk of Mobile Nucleic Acids in Parasitic Plants. Nat. Plants 5 (9), 991–1001. 10.1038/s41477-019-0458-0 31332314

[B170] Yang Z. Zhang Y. Wafula E. K. Honaas L. A. Ralph P. E. Jones S. (2016). Horizontal Gene Transfer Is More Frequent with Increased Heterotrophy and Contributes to Parasite Adaptation. Proc. Natl. Acad. Sci. U. S. A. 113 (45), E7010–E7019. 10.1073/pnas.1608765113 27791104 PMC5111717

[B171] Yin Z. Zhu B. Feng H. Huang L. (2016). Horizontal Gene Transfer Drives Adaptive Colonization of Apple Trees by the Fungal Pathogen *Valsa mali* . Sci. Rep. 6, 33129. 10.1038/srep33129 27634406 PMC5025739

[B172] Yonemochi C. Suga K. Harada C. Hanazumi M. (2010). Tevaluation of Transgenic Event CBH 351 (StarLink) Corn in Pig. Animal Sci. J. 81 (1), 94–101. 10.1111/j.1740-0929.2009.00718.x 20163679

[B173] Yoshida S. Cui S. Ichihashi Y. Shirasu K. (2016). The Haustorium, a Specialized Invasive Organ in Parasitic Plants. Annu. Rev. Plant Biol. 67 (1), 643–667. 10.1146/annurev-arplant-043015-111702 27128469

[B174] Zhao K. Ren F. Han F. Liu Q. Wu G. Xu Y. (2016). Edible Safety Assessment of Genetically Modified Rice T1C-1 for Sprague Dawley Rats through Horizontal Gene Transfer, Allergenicity and Intestinal Microbiota. PLOS ONE 11 (10), e0163352. 10.1371/journal.pone.0163352 27706188 PMC5051820

[B175] Zhu B. (2006). Degradation of Plasmid and Plant DNA in Water Microcosms Monitored by Natural Transformation and Real-Time Polymerase Chain Reaction (PCR). Water Res. 40 (17), 3231–3238. 10.1016/j.watres.2006.06.040 16945402

[B176] Zhu B. Lou M.-M. Xie G.-L. Zhang G.-Q. Zhou X.-P. Li B. (2011). Horizontal Gene Transfer in Silkworm, *Bombyx mori* . BMC Genomics 12 (1), 248. 10.1186/1471-2164-12-248 21595916 PMC3116507

[B177] Zhu Y. Li D. Wang F. Yin J. Jin H. (2004). Nutritional Assessment and Fate of DNA of Soybean Meal from Roundup Ready or Conventional Soybeans Using Rats. Archives Animal Nutr. 58 (4), 295–310. 10.1080/00039420412331273277 15570744

